# Recent advances in heteroatom-doped graphene quantum dots for sensing applications

**DOI:** 10.1039/d1ra04248c

**Published:** 2021-07-23

**Authors:** Neeraj Sohal, Banibrata Maity, Soumen Basu

**Affiliations:** School of Chemistry and Biochemistry, Thapar Institute of Engineering and Technology Patiala 147004 India banibrata.maity@thapar.edu soumen.basu@thapar.edu

## Abstract

Graphene quantum dots (GQDs) are carbon-based fluorescent nanomaterials having various applications due to attractive properties. But the low photoluminescence (PL) yield and monochromatic PL behavior of GQDs put limitations on their real-time applications. Therefore, heteroatom doping of GQDs is recognized as the best approach to modify the optical as well as electronic properties of GQDs by modifying their chemical composition and electronic structure. In this review, the new strategies for preparing the heteroatom (N, B, S, P) doped GQDs by using different precursors and methods are discussed in detail. The particle size, emission wavelength, PL emissive color, and quantum yield of recently developed heteroatom doped GQDs are reported in this article. The investigation of structure, crystalline nature, and composition of heteroatom doped GQDs by various characterization techniques such as high-resolution transmission electron microscopy (HRTEM), X-ray diffraction (XRD), Raman spectroscopy, Fourier transform infrared (FTIR) and X-ray photoelectron spectroscopy (XPS) are also described. The recent progress on the impact of mono or co-doping of heteroatoms on PL behavior, and optical, electrochemiluminescence (ECL), and electrochemical properties of GQDs is also surveyed. Further, heteroatom doped GQDs with attractive properties used in sensing of various metal ions, biomolecules, small organic molecules, *etc.* by using various techniques with different limits of detection are also summarized. This review provides progressive trends in the development of heteroatom doped GQDs and their various applications.

## Abstract 2

Graphene quantum dots (GQDs) are carbon-based fluorescent nanomaterials having various applications due to attractive properties.

## Graphical abstract

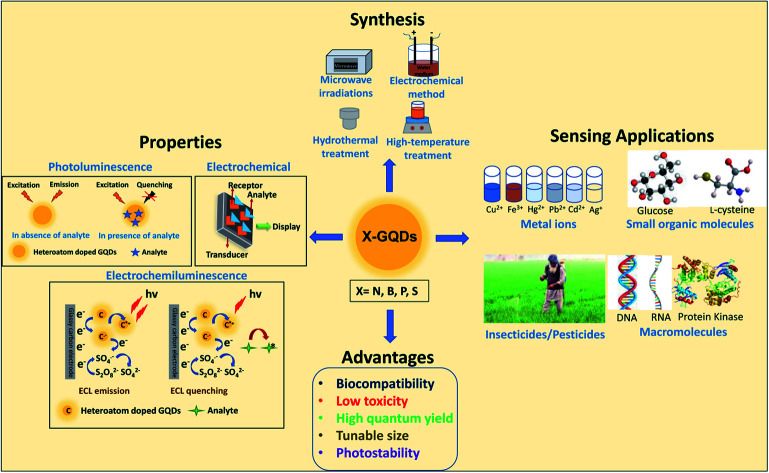

## 1. Introduction

Graphene is a mono-layer carbon nanostructure in which carbon atoms are assembled in a two-dimensional (2D) honeycomb crystal lattice.^1^ Graphene has had various applications due to its unique chemical, thermal, mechanical, and physical properties since its discovery in 2004.^2^ These properties can be improved by manipulating the shape, size, and sp^2^-hybridized carbon domains using redox reactions.^3^ Photoluminescence (PL) is not observed in the case of graphene due to its zero band gap which makes it an unsuitable material for optoelectronics and electronics applications. Therefore, there is a need to resolve the problem of zero-band gap materials and it can be solved by altering 2D graphene into zero-dimensional (0D) graphene quantum dots (GQDs).^1,4^

Since its discovery in 2008, GQDs are a new type of carbon-based fluorescent nanomaterial that have attained great attention in research areas due to their attractive properties.^5^ GQDs consist of highly crystalline graphene sheets having dimensions below 100 nm in one-, two- and few-layers (>10 layers).^6^ GQDs' structure has a thickness close to the dimensions of a single atom.^7^ Generally, GQDs contain sp^2^ or sp^3^ carbon atoms and functional groups based on oxygen. GQDs possess extraordinary properties, a size-dependent and non-zero electronic band gap as compared to bulk graphene. GQDs are designed when there is a fragmentation of graphene sheets into less than the exciton Bohr radius (20 nm).^8^ GQDs have exceptional properties such as PL, multi-color emission, brilliant photostability, biocompatibility, electrochemiluminescence (ECL), chemical inertness, small size derived from the quantum confinement effect (QCE), and edge effects. The carboxylic groups located at the edges of GQDs enhance their water solubility as well as providing the potential for functionalization with many inorganic, organic, polymeric, and biomolecules. These extraordinary properties of GQDs lead to enhancement of their applications in various fields such as energy storage,^9^ biosensing,^10,11^ bioimaging,^12^ drug delivery,^13^ photocatalysis,^14^ and many others. GQDs act as electron donors and acceptors owing to their great surface area and a huge number of edge sites. These properties increase the ECL and electrochemical activity of GQDs which further enhances their application in biosensing. In recent years, different mechanisms were reported for the PL emission of GQDs including QCE, recombination of localized electron–hole pairs, the zig–zag edge sites effect, the electronegativity of heteroatoms, and surface defects.^15,16^ GQDs can be synthesized by mainly two approaches *i.e.*, the “top-down” approach and the “bottom-up” approach. In recent years, many researchers have synthesized GQDs by different methods under these two approaches.

The two contradictory PL emission behaviors *i.e.*, excitation-independent and excitation-dependent found in GQDs and their exact mechanism are still under debate. Different luminescence colors can be attained by tuning the excitation wavelength or concentration of GQDs.^16^ By regulating size, shape, edge configuration, defects, functional groups, as well as heterogeneous hybridization of the carbon lattice, the PL emission of GQDs can be extensively changed from the deep ultraviolet region to the near-infrared region.^17^

In recent years, GQDs have been utilized as a great sensing platform for metal ions/biomolecules detection as compared to other standard methods. But the detection sensitivity decreases due to the poor quantum yield of GQDs. Therefore, surface functionalization and doping with heteroatoms such as nitrogen (N), sulfur (S), boron (B), and phosphorous (P) enhance the quantum yield of GQDs.^18^ Surface functionalization of GQDs through organic molecules and polymers occupies the specific functional positions on the GQDs which makes them selective for the sensing of metal ions and biomolecules. Heteroatom doped GQDs improve the physical as well as chemical properties such as chemical reactivity, optical activity, and electronic structure of GQDs by tuning their intrinsic properties.^19,20^ Hence, there is an urgent need to study the functionalized and heteroatom doped GQDs as they have great potential in biosensing.

## 2. Synthesis of heteroatom doped GQDs

Generally, the synthesis of GQDs consists of two main strategies *i.e.*, “top-down” and “bottom-up” strategies.^21^ The “top-down” method include cutting or breaking of carbonaceous materials, *e.g.*, graphene, carbon nanotubes (CNTs), graphite, graphene oxide (GO), carbon fibers, carbon black by using different methods including hydrothermal treatment, electrochemical exfoliation, oxidation by strong acids, and many others.^22^ The “top-down” strategy produces high-quality GQDs at a large scale but this strategy possess various limitations due to non-uniform product size, low product yield, harsh synthesis process, and requirement of special equipment. However, the bottom-up strategy includes the preparation of GQDs by using small molecular fragments through thermal or catalytic treatment. This strategy has good control on the intrinsic properties of the product like size, lattice dimensions, morphology, and results in the production of a large number of GQDs with eco-friendly nature.^19^ In the bottom-up approach small molecules undergo both dehydrogenation and carbonization processes in the hydrothermal/solvothermal method and result in higher efficiency as compared to reported values.^23^ To enhance the intrinsic properties of GQDs, different heteroatom doped GQDs were prepared by both “top-down” and “bottom-up” approaches. These approaches for the preparation of doped GQDs are further divided into two categories which include the multi-step method and the one-step method. In multi-step methods, first step is to prepare GO, second step is to reduce GO to reduced GO (rGO), then synthesis of GQDs, and further heteroatom doping can be achieved by using the electrochemical method, hydrothermal treatment, or pyrolysis. Among these methods, it is further require the cutting of doped graphene into doped GQDs by chemical oxidation method.^24,25^ In the one-step method, there is one pot reaction between carbon source and heteroatom source by hydrothermal method, high-temperature treatment, microwave irradiations, or chemical vapor deposition (CVD). The major requirement of one-step methods is the presence of a doping source in the starting material to obtain doped GQDs. Recently, researchers prefer to follow the one-step method with a “bottom-up” approach to prepare doped GQDs as compared to the multi-step method due to the time-consuming and complicated procedure.

There are different review articles available on the graphene quantum dots,^4^ heteroatom-doped carbon quantum dots,^26^ and heteroatom-doped graphene,^27^ but not specifically on the heteroatom-doped graphene quantum dots. Some reports discussed the general synthesis, properties, and application of GQDs. But in this article, we have discussed the synthesis of single and co-doped GQDs and their structural properties in detail. The detail investigation of different characterizations of heteroatom doped GQDs also reported here. Moreover, we have discussed the impact of heteroatom doping on the properties of GQDs and applications of heteroatom-doped GQDs as a sensor with their advantages and disadvantages. So, this review article will give all the detail information about the heteroatom-doped GQDs.

### 2.1. Synthesis of nitrogen-doped GQDs (N-GQDs)

**Fig. 1:**
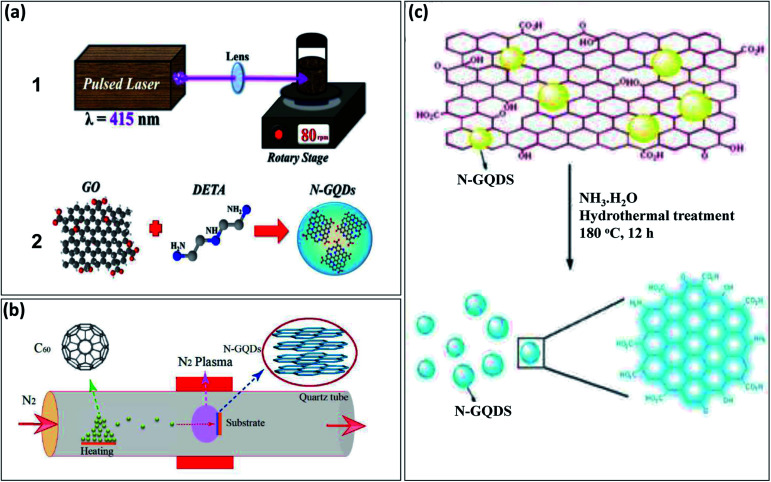
Schematic representation of different methods to prepare N-GQDs by “top-down” approach: (a1) experimental set-up based on pulse laser ablation method, (a2) synthesis process by using GO and DETA,^28^ (b) nitrogen plasma treatment method performed in quartz chamber with 2.45 GHz microwave radiation,^29^ and (c) hydrothermal treatment of GO with ammonia solution.^30^ Part (a1) and (a2) are reproduced from ref. 28 with permission from [PCCP Owner Societies], copyright [2017], part (b) is reproduced from ref. 29 with permission from [American Chemical Society], copyright [2018], and part (c) is reproduced from ref. 30 with permission from [Royal Society of Chemistry], copyright [2013].

Recently, different approaches have been reported for the development of N-GQDs through both “top-down” and “bottom-up” approaches. The “top-down” approach includes hydrothermal, microwave irradiations, pulse laser ablation, and electrochemical treatment of carbon sources, such as GO, rGO, C_60_, and CNTs with a nitrogen source such as ammonia solution. Santiago *et al.*^28^ used the pulse laser ablation (PLA) technique displayed in Fig. 1a1 for the preparation of N-GQDs in which GO was used as a carbon source and diethylenetriamine (DETA) as a nitrogen source and its synthesis process represented in Fig. 1a2. Zheng *et al.*^29^ prepared N-GQDs *via* the CVD technique in which C_60_ powder was converted into C_60_ molecules by evaporating up to its sublimation temperature and C_60_ molecules further passed through the plasma zone in the presence of nitrogen gas. The reaction between active C_60_ and nitrogen plasma results in the formation of N-GQDs on a nickel foil substrate (Fig. 1b). Hu *et al.*^30^ developed N-GQDs by using GO present in ammonia solution by one-step hydrothermal process and schematic diagram of its synthesis process represented as in Fig. 1c. These methods require special equipment and are slightly complicated. Therefore, the one-step method with a “bottom-up” approach is most preferable to prepare N-GQDs due to less time-consuming and easy approach.

**Fig. 2:**
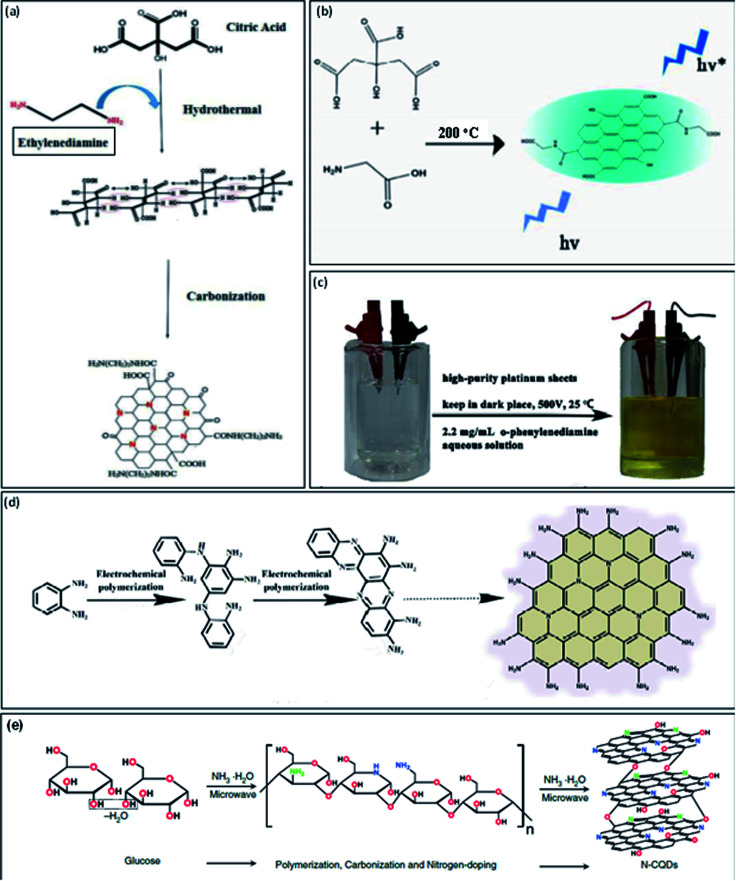
Schematic representation of one-step methods to prepare N-GQDs by “bottom-up” approach: (a) growth model from CA and ethylenediamine by using a hydrothermal method,^31^ (b) schematic illustration by high-temperature treatment,^25^ (c) digital image for the electrochemical set-up including *o*-phenylendiamine aqueous medium (left) and the product obtained after the completion of the reaction (right),^32^ (d) schematic diagram of the reaction mechanism of electropolymerization process^32^ and (e) synthesis process from glucose in ammonia solution by using microwave irradiations.^24^ Part (a) is reproduced from ref. 31 with permission from [Elsevier], copyright [2017], part (b) is reproduced from ref. 25 with permission from [Royal Society of Chemistry], copyright [2016], part (c) and (d) are reproduced from ref. 32 with permission from [Royal Society of Chemistry], copyright [2016] and part (e) is reproduced from ref. 24 with permission from [De Gruyter], copyright [2017].

Recently, N-GQDs have been also prepared by using different carbon sources such as citric acid (CA), glucose, or glycine, and nitrogen sources such as ammonia, glycine, dicyandiamide (DCD), tris(hydroxymethyl) aminomethane, or 3,4-dihydroxy-l-phenylalanine, by using the hydrothermal method, microwave irradiations, or pyrolysis.^24^ These methods undergo main processes such as polymerization, carbonization, and further doping with nitrogen heteroatom in a single reaction for developing N-GQDs. Safardoust-Hojaghan *et al.*^31^ synthesized N-GQDs by using CA along with ethylenediamine under hydrothermal treatment. The hydrothermal treatment leads to the first carbonization of starting materials and then further converted into N-GQDs (Fig. 2a). The water-soluble N-GQDs have been developed through one-step high-temperature treatment. The CA (carbon precursor) and glycine (nitrogen precursor) undergo pyrolysis at 200 °C for the development of N-GQDs. The schematic illustration for the formation of N-GQDs is shown in Fig. 2b.^25^

Tian *et al.*^32^ prepared N-GQDs with large-scale –NH_2_ edge groups by using the “bottom-up” electrochemical method. They used only *o*-phenylenediamine (reactant) in an aqueous medium (electrolyte) and two highly pure platinum sheets as anode and cathode in this electrochemical method (Fig. 2c). They used dark conditions to remove the risk of spontaneous oxidation of *o*-phenylenediamine. Fig. 2d represents the formation mechanism of N-GQDs in which *o*-phenylenediamine is coupled together to form amine bonds. Further, the oxidative coupling occurs between partial amino groups and amine bonds which result in the development of phenazine structure. At the end of the reaction, there was the formation of N-GQDs with precise amino edge groups. There were several methods reported for the formation of N-GQDs but microwave irradiations provide fast and strong heating which completes the long hour reactions in few minutes. Zheng *et al.*^24^ used one-step microwave irradiations to develop N-GQDs in which glucose used as carbon precursor and ammonia as nitrogen precursor. They found that the ammonia used for the formation of N-GQDs was also acts as a catalyst for the dehydration reaction among molecules of glucose and water medium. Microwave heating enhances the reaction rate by providing rapid heat for glucose polymerization and carbonization and results in the fast formation of N-GQDs within 1 min. Fig. 2e represents the formation mechanism of N-GQDs. There were various one-step methods reported in the literature for the development of N-GQDs with variable sizes, PL emissive colors, and quantum yields are mentioned in Table 1.

**Table 1:** Synthesis & purification methods of heteroatom doped GQDs and their corresponding properties

Hetero-atom doped GQDs	Precursor	Synthesis & purification	Method	Size (nm)	*λ*_ex_ (nm)/*λ*_em_ (nm)	QY (%)	Ref. & (year)
N-GQDs	GO + ammonia	The mixture of GO (dispersed in water) and ammonia was heated at 180 °C for 12 h using the hydrothermal method and then filtered	Hydrothermal treatment	3.5	340/436	24.6	30 & (2012)
		The supernatant was dialyzed by a dialysis bag (3000 Da) for 2 h					
	CA + hydrazine	(1) Pyrolyzing of CA by heating at 200 °C for 30 min	Multi-step hydrothermal treatment	3.8	360/440	23.3	46 & (2014)
		(2) Hydrothermal treatment of prepared solution at 180° for 12 h					
		Centrifuge for 10 min					
	CA + dicyandiamide	CA and dicyandiamide dissolved in water were heated at 180 °C for 3 h	Hydrothermal method	2.3	370/452	36.5	47 & (2014)
		The solution was centrifuged (12 000 rpm) for 15 min and then dialysis membrane used (1000 Da) for 48 h					
	CA + ammonia	Carbonization of CA with NH_3_ by heating at 200 °C for 3 h *via* hydrothermal method	Hydrothermal treatment	3.5	325/420	30.7	19 & (2014)
		A dialysis bag of 3000 Da was used for 4 h					
	CA + glycine	The mixture of CA and glycine was heated at 200 °C for 5 min and an orange solution formed	High-temperature treatment	1–4	355/450	28.1	25 & (2016)
		No purification steps					
	Glucose + ammonia	Glucose in distilled water with ammonia was heated at 900 W for 1 min through a commercial microwave oven	Microwave irradiation	5.3	360/430	6.4	24 & (2016)
		The solution was kept at 40 °C for 1 h and further, purified by dialysis membrane (1000 Da)					
	*o*-Phenylene-diamine	*o*-Phenylenediamine was used as a reactant in water as an electrolyte. In this electrochemical process, 500 V was applied to the two Pt electrodes under dark conditions for 1 h	Electrochemical method	3.9	420/569	71.0	32 & (2016)
		No purification steps					
	CA + ethylenediamine	The solution of equal ratio of CA and ethylenediamine was heated at 180 °C for 6 h	Hydrothermal treatment	15–20	330/565	25.0	31 & (2017)
		No purification steps					
	Chitosan	Firstly, chitosan gets decomposed into N-compound and further HCN gets adsorbed on the copper's surface and then nucleation	Chemical vapor deposition (CVD)	10–15	333/448	—	48 & (2018)
		No purification steps					
	C_60_/nitrogen plasma	The transformation of C_60_ molecules in a nitrogen plasma heated at 440 °C through microwave by using nickel foil as substrate	Plasma treatment by (CVD) technique	4.5	336 to 392/412 & 432	7.4	29 & (2018)
		No purification steps					
	N-CNT/N-graphene in ammonia solution	N-CNT/N-graphene as the working electrode and the platinum sheet as counter electrode and ammonia solution as an electrolyte used in the two-electrode electrochemical system and 0.01 A charging current was supplied for 4–8 h	Electro-chemical method	2.0 ± 0.5	380/455	19.3	49 & (2019)
		Membrane filter with a pore size of 0.22 μm					
B-GQDs	Boron-doped graphene (BG)	First step, oxidation of BG to oxidized BG (BGO) by heating at 300 °C for 2 h in presence of HNO_3_ (40%) *via* tube furnace at inert atmosphere	Hydrothermal treatment	2 to 4	310/440	—	50 & (2014)
		Second step, oxidation of BGO in presence of H_2_SO_4_ and HNO_3_ (1 : 3) for 17 h under ultrasonication. After adjusting the pH to 8, the solution was heated at 200 °C for 11.5 h					
		The pale-yellow solution was filtered by a dialysis bag of 3500 Da for 12 h					
	Graphite rod in 0.1 M borax	The voltage of 3 V was applied for 2 h at graphite rod (anode) dipped in 0.1 M borax and Pt sheet (cathode) undergo electrolysis	Electrolyzing method	4.5	360/530	9.3	34 & (2016)
		The prepared solution was filtered by 0.22 μM and then dialyzed with a dialysis bag (3500 Da) for 48 h					
	Boron-doped graphene rods	B-doped graphene rods as anode and graphite rods as a cathode in the mixture of ethanol and water with 99.5 : 0.5 as electrolyte was used in an electrochemical process. The current intensity of 200–250 mA cm^−2^ for 2 h was applied for electrochemical reaction	Electrochemical method	5 to 10	420/530	—	33 & (2016)
		The resultant solution was centrifuged at 9000 rpm and purified by a dialysis bag of 1000 for 24 h					
	VPBA (4-vinylphenylboronic acid) and boric acid	The sonication of VPBA and boric acid dispersed in acetone and ethanol for 30 min and further addition of H_2_O_2_. The solution was heated at 205 °C for 24 h	High-temperature treatment	5.8	360/445	11.2	37 & (2016)
		The prepared solution was centrifuged for 20 min at 20 000 rpm for 3 cycles and dialyzed by dialysis bag (12 000–14 000 Da) for 3 days					
	1,3,6-Trinitropyrene and borax in NaOH	Ultrasonication of 1,3,6-trinitropyrene and borax dissolved in NaOH solution for 30 min and heated at 200 °C for 6 h through hydrothermal treatment	Hydrothermal treatment	2	480/520	16.8	35 & (2019)
		The prepared solution was dialyzed by a dialysis bag (500 Da and 3500 Da)					
	Boron carbide	Firstly, boron carbide converted to boron-doped graphene (BG) by heating at 1400 °C for 3 h	Microwave irradiation	6 ± 2	320/420 and 440	—	38 & (2019)
		Then, BG dispersed in polyethylene glycol heated at 220 °C for 30 min by microwave reactor					
		The formed solution was dialyzed through a dialysis bag (14 000 Da) for 2 days					
S-GQDs	Graphite in sodium *p*-toluenesulfonate aqueous solution	Graphite rod as working electrode dipped in sodium *p*-toluenesulfonate aqueous solution and Pt foil as the counter electrode was used in electrochemical process under voltage of 5 V for 3 h	Electrolyzing method	3	380/480	10.6	43 & (2014)
		Filtered by 0.22 μM membrane and then, dialyzed by dialysis bag (3500 Da) for 1 day					
	1,3,6-(Trinitropyrene + NaOH + Na_2_S)	The ultrasonication of 1,3,6-trinitropyrene dispersed in NaOH solution with Na_2_S for 1 h. Then, the solution was heated at 200 °C for 10 h	Hydrothermal treatment	3	490/535	11.6	41 & (2016)
		Filtered by 1000 Da dialysis bag for 2 days and then by 3500 Da for 1 day					
	3-Mercaptopropionic acid (MPA)	Ultrasonication of a mixture of 1,3,6-trinitropyrene and MPA for 1 h. Further, the solution was heated at 200 °C for 10 h *via* hydrothermal method	Hydrothermal treatment	2.5	360/450	9.2	42 & (2017)
		Filter through 0.22 mm microporous membrane. Then, purified by dialysis bag of 3500 Da and 1000 Da for 24 h each					
	CA + powdered S	CA and powdered S were dispersed in distilled water and then, heated at 170 °C for 4 h in an electric oven	Hydrothermal treatment	3.93	380/460	—	44 & (2018)
		The prepared solution was centrifuged (5000 rpm) for 5 min					
	Green extract (sugarcane molasses)	Sugarcane molasses was stirred for 2 h and heated at 180 °C for 4 h	Hydrothermal treatment	3.5 ± 1.25	380/515	47.0	45 & (2018)
		The prepared black crude product was washed with water and filtered with many grade Gooch crucibles					
P-GQDs	Sodium phytate	The graphite rod as a working electrode and Pt electrode as a counter electrode used in an electrochemical process and sodium phytate solution as electrolyte. The voltage of 5 V was applied for 12 h	Electrochemical method	2–4	—	—	39 & (2017)
		Filtered by 220 nm filter and purified by a dialysis bag (3500 Da) for six days					

### 2.2. Synthesis of boron-doped GQDs (B-GQDs)

In the periodic table, B is a neighbor element of carbon and has slightly similar atomic size as compared to carbon.^33^ The difference in the electronegativity between C (2.55) and B (2.04) can result in considerable charge transfer in C–B composites. B-doping is also called hole doping as it causes defects in the framework of GQDs. Also, doping of B on the surface of GQDs leads to efficiently modify the electronic nature of GQDs and have unique characteristics.^34,35^ There have been very few publications reported on the synthesis and application of B-GQDs as compared to N-GQDs, due to difficulties in the synthesis process of B-GQDs.^36^ Some reported synthesis methods of B-GQDs include cutting of boracic graphite through the electrolyzing method, glucose carbonization in presence of boric acid, cutting of B-doped graphene by strong acids, high-temperature treatment of GO in Na_2_B_4_O_7_ solution, and decomposition of B-doped graphene rods in the presence of organic solvent by microwave treatment.^35^

**Fig. 3:**
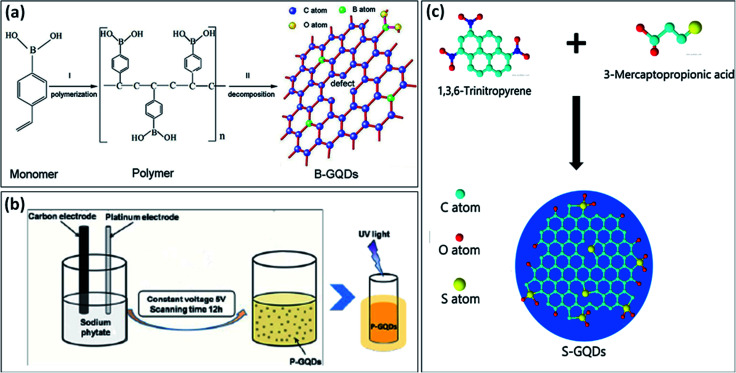
(a) stepwise formation mechanism of B-GQDs,^37^ (b) schematic diagram representing the synthesis of P-GQDs and its yellow emission under UV light (365 nm),^39^ and (c) schematic diagram showing the synthesis process of S-GQDs using bio-waste.^42^ Part (a) is reproduced from ref. 37 with permission from [Wiley Online Library], copyright [2017], part (b) is reproduced from ref. 39 with permission from [Royal Society of Chemistry], copyright [2017], and part (c) is reproduced from ref. 42 with permission from [Elsevier], copyright [2017].

Wang *et al.*^37^ used 4-vinylphenylboronic acid (VPBA) and boric acid under high-temperature treatment (200 °C) for the formation of B-GQDs. The two steps were involved in the formation of B-GQDs (Fig. 3a); the first step includes polymerization of VPBA molecules under high pressure and temperature and decomposition of H_2_O_2_ to form free radicals *i.e.*, hydroxyl (HO˙) and hydroperoxyl (HOO˙) radicals. In the second step, the C–H bond in the benzene ring and O–B bond in boric acid were ruptured to produce B-doped carbon-based free radicals and further form bigger carbon-based fragments. The defects in the framework of GQDs enhance the active sites for the B-doping. Chen *et al.*^34^ synthesized B-GQDs by means of graphite rod in borax aqueous solution through the electrolyzing method. Ge *et al.*^35^ used a one-step hydrothermal method under the “bottom-up” strategy for the synthesis of B-GQDs through molecular fusion of 1,3,6-trinitropyrene and borax in NaOH solution. Vijaya *et al.*^38^ prepared B-GQDs by first converting bulk boron carbide (B_4_C) crystals into B-doped graphene (BG) followed by the uniform cutting of BG by using a microwave reactor. Tam *et al.*^36^ used a facile one-step hydrothermal approach at low temperature for the synthesis of B-GQDs by using glucose and boric acid. They prepared different types of B-GQDs with the variation in the B content and found that highly doped B-GQDs (up to 4.25%) showed exceptional electrocatalytic properties. They found an efficient method for preparing highly doped B-GQDs by using controlling the concentration of B. Zhang *et al.*^33^ used the electrochemical method for the formation of B-GQDs from a mixture of heteroatom salt in electrolyte and graphite rods as both cathode and anode. They prepared B-GQDs by electrolytic exfoliation of B-doped graphene rods which increase the uniformity of the B-GQDs.

### 2.3. Synthesis of phosphorous-doped GQDs (P-GQDs) and sulfur-doped GQDs (S-GQDs)

There have been very few methods reported for the synthesis of P-doped GQDs because lattice doping of P-GQDs is very difficult due to different oxidation states of P. Li *et al.*^39^ reported an electrochemical method for the synthesis of P-GQDs in which sodium phytate (C_6_H_6_Na_12_O_24_P_6_) acts as an electrolyte which offers the phosphorous content. The schematic diagram in Fig. 3b, describes the synthesis process for the yellow emissive P-GQDs by using an electrochemical approach. Wang *et al.*^40^ utilized a green approach to prepare P-GQDs by solvothermal treatment of lecithin. They found that lattice doping of GQDs was successfully obtained by the efficient *in situ* reduction of lecithin. But still, there is a need to advance the methods for the preparation of P-GQDs.

Among different heteroatoms, S doping is distinct due to differences in the outmost orbitals of S and C makes an on-uniform spin density distribution that results in providing S-doped materials that show exceptional properties.^41^ S-atom is larger as compared to a carbon atom and the bond length of the C–S bond is 1.78 Å which is 25% greater in length as compared to the C–C bond. The electronegativity difference among S (2.58) and C (2.55) is very less which results in insignificant polarization or transfer of charge in the C–S bond. Therefore, S-doping into the structure of GQDs is quite hard due to difficulty in the formation of the C–S bond.^41,42^ Li *et al.*^43^ used an electrochemical method for the synthesis of S-GQDs through the electrolysis process of graphite present in sodium *p*-toluene sulfonate aqueous medium. Mostly, the hydrothermal treatment has been utilized for the development of S-GQDs as compared to the other methods. Bian *et al.*^41^ used a one-step hydrothermal reaction for the formation of S-GQDs based on aqueous-phase molecular fusion by using 1,3,6-trinitropyrene, Na_2_S, and NaOH. Bian *et al.*^42^ utilized 6-trinitropyrene as a carbon precursor and 3-mercaptopropionic acid (MPA) as a sulfur precursor for the development of S-GQDs through a hydrothermal approach (Fig. 3c). They found that the prepared S-GQDs have one layer graphene structure, uniformity, and exhibit strong blue emission. Jin *et al.*^44^ developed a simple one-step hydrothermal approach for preparing S-GQDs by using CA and powdered sulfur. They found that the prepared S-GQDs showed strong blue emission and have good water solubility as compared to undoped GQDs. Sangam *et al.*^45^ reported a hydrothermal approach for developing S-GQDs by using one precursor *i.e.*, second generation green agro-industrial wastes. They developed a fast and eco-friendly method for the preparation of S-GQDs in which there was no requirement of catalysts or organic solvents.

### 2.4. Synthesis of co-doped GQDs

**Fig. 4:**
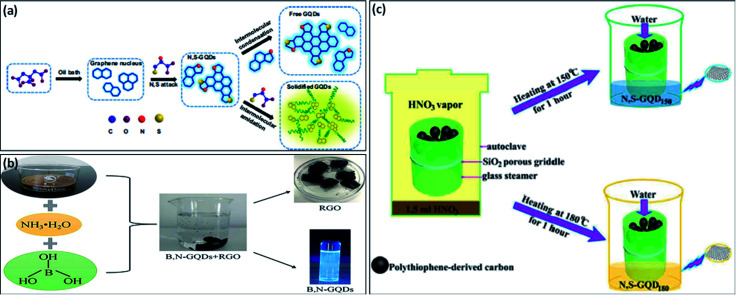
(a) Schematic diagram for the synthesis of free N, S-GQDs, and solidified GQDs,^22^ (b) preparation of B, N-GQDs by one-pot hydrothermal method,^54^ and (c) schematic diagram of developing N, S-GQDs with tunable luminescence.^57^ Part (a) is reproduced from ref. 22 with permission from [Elsevier], copyright [2017], part (b) is reproduced from ref. 54 with permission from [Elsevier], copyright [2020], and part (c) is reproduced from ref. 57 with permission from [Royal Society of Chemistry], copyright [2016].

Recently, many researchers have significantly varied the electronic structure and optical properties of GQDs by mono-doping or co-doping non-metallic elements.^51^ Co-doped GQDs with heteroatoms like N, B, S, and P improve the quantum yield of undoped or mono-doped GQDs by generating additional coordination sites and providing more defects in the structure of prepared GQDs.^52^ It was reported that heteroatom doped GQDs with large size atoms like N, S, or small size atoms like P, B as compared to C showed exceptional properties.^20^ Qu *et al.*^20^ prepared multi-emissive S, N-GQDs by using CA and thiourea as precursors through the solvothermal method. They reported that, CA serves as a carbon precursor and thiourea serves as both nitrogen and sulfur precursor. They used *N*,*N*-dimethylformamide (DMF) as a solvent as well as passivating agent and result in a high percentage of N in prepared S, N-GQDs. Shen *et al.*^53^ developed S, N-GQDs by using a one-step hydrothermal technique in which molecular fusion occurs among precursors used *i.e.*, 1,3,6-trinitropyrene, thiourea, DMF, and sodium hydroxide. Xia *et al.*^22^ developed two types of GQDs which include free N, S-doped GQDs, and solidified GQDs in which CA is used as precursor and l-cysteine is used as a dopant through solvent-free “bottom-up” approach (Fig. 4a). They reported that, firstly graphene nucleus was formed due to the dehydration of CA and then, attack of N, S atoms present in l-cysteine on the nucleus of graphene results in the incorporation of these atoms in the framework of graphene. Free N, S co-doped GQDs was also formed by further crosslinking of developed graphene nucleus through an intermolecular condensation reaction. Furthermore, the solidified N, S co-doped GQDs were formed with the formation of acylamino oligomer between l-cysteine and nucleus of graphene.

The chemical doping of N as electron doping or B as hole doping into the structure of GQDs is easy due to quite similar atomic size. The electronegativity difference between C (2.55) and N (3.04)/B (2.04) is sufficient for the charge transfer in the C–N/C–B bond.^34^ Liu *et al.*^51^ described a rapid and facile process for the synthesis of B, N doped GQDs in which there was no requirement of any strong acid and surface deactivator. They prepared B, N-GQDs through pyrolysis in which CA, urea, and boric acid were used as precursors. Liu *et al.*^54^ used a “top-down” strategy for the synthesis of B, N-GQDs. They used GO, boric acid, and ammonia solution as precursors for the preparation of B, N-GQDs *via* a hydrothermal method (Fig. 4b).

The growth of co-doped GQDs with electron-rich N atom has forced to prepare the other new co-doped GQDs with electron-rich atoms, like P and S. Both P and S atoms have greater atomic radii as compared to carbon atoms and also the difference in the electronegativity of P (2.19), S (2.58), and C (2.55) is very less which result as insignificant charge transfer in C/P and C/S. Co-doping of GQDs into its structure with these elements was a quite difficult process and therefore, co-doped GQDs were exceptionally reported.^55^ Peng *et al.*^56^ described an electrochemical technique in which graphite electrode (C source), sodium phytate (P source), and sodium sulfide (Na_2_S) (S source) were used for the formation of S, P-GQDs. It was reported that the PL quantum yield of S, P-GQDs was greater than mono-doped GQDs *i.e.*, S-GQDs and P-GQDs.^55^ Anh *et al.*^18^ reported a one-step hydrothermal approach for preparing N, S co-doped GQDs by using CA and thiourea. They found a high quantum yield of prepared GQDs in comparison with undoped GQDs. Xu *et al.*^57^ prepared two types of N, S co-doped GQDs by using polythiophene derived carbon source at different two temperatures. They found that two types of prepared GQDs showed different fluorescence and different properties due to variation in particle size as well as different surface defects. Fig. 4c represents the schematic presentation of the synthesis of N, S co-doped GQDs with different luminescence. Liu *et al.*^58^ formed N, P co-doped GQDs by using tetrakis(hydroxymethyl) phosphonium chloride (THPC) as carbon as well as phosphorous source and ethylenediamine endcapped polyethylenimine (PEI-EC) as nitrogen source and also passivation agent through hydrothermal treatment. Kundu *et al.*^59^ synthesized S, N, F co-doped GQDs by using multiwalled CNTs (MWCNTs) in ionic liquid *i.e.* 1-methyl-1-propylpiperidinium bis (trifluoromethylsulfonyl)imide through microwave treatment. They reported that microwave heating enhances the rate of the reaction with a high yield as compared to other conventional methods by the reduction of GO. The ionic liquid with exceptional chemical structure provides the efficient co-doping by N, S, and F heteroatoms. They also reported that, MWCNTs act as carbon source and the ionic liquid act as a strong source for N, S, and F in the preparation of N, S, F co-doped GQDs. Xu *et al.*^52^ developed N, P, S co-doped GQDs from anthracite coal (raw material) by using one-step wet chemical dialysis method. They reported an easy and cheap process for the preparation of N, P, S-GQDs. The different types of co-doped GQDs with synthesis methods, quantum yields, excitation and emission wavelength are tabulated in Table 2.

**Table 2:** Synthesis & purification methods of heteroatom co-doped GQDs and their corresponding properties

Co-doped GQDs	Precursor	Synthesis & purification	Method	Size (nm)	*λ*_ex_ (nm)/*λ*_em_ (nm)	QY (%)	Ref. & (year)
N, F, S-GQDs	MWCNTs in a customized ionic liquid medium	MWCNTs dispersed in an ionic liquid by sonicating it for 1 h and then, kept for 15 min in a microwave (1100 W)	Microwave treatment	2.0	375/409 and 435	70.0	59 & (2015)
		Filtered by 0.2 μm polytetrafluoroethylene (PTFE) membrane and dialyzed for 7–8 h					
N, S-GQDs	1,3,6-Trinitropyrene + thiourea + DMF + NaOH	1,3,6-Trinitropyrene and thiourea were dispersed in NaOH solution with DMF and sonicated for 30 min. The solution was heated at 200 °C for 10 h by using the hydrothermal method	Hydrothermal treatment	2.1	375/450	23.2	53 & (2017)
		Filtered by a dialysis bag of 1000 Da for 1 day					
N, S-GQDs	CA + thiourea	CA and thiourea were dispersed in distilled water and then heated at 160 °C for 4 h	Hydrothermal treatment	3.5 ± 0.5	340/445	41.9	18 & (2017)
		Centrifuged at 5000 rpm for 5 min					
B, N-GQDs	*p*-Phenylenediamine + formylphenylboronic acid	*p*-Phenylenediamine and formylphenylboronic acid were dispersed in ethanol and the solution was heated hydrothermally at 180 °C for 24 h	Solvent thermal method	8.1 ± 1.2	400/560	—	60 & (2017)
		Filtered by 0.22 μm membrane and dialyzed for 48 h					
N, S-GQDs	CA + l-cysteine	The mixture of CA and l-cysteine in a round bottom flask was heated at 240 °C for 2 min and the orange color solution was obtained in 4 min	High temperature pyrolysis	2.8 ± 1.0	350/430	84.0	61 & (2017)
		Filtered by a dialysis bag of 1000 Da for 8 h					
N, P, S-GQDs	Anthracite coal	Anthracite coal was dispersed in H_2_SO_4_ and HNO_3_ (3 : 1) and sonicated for 2 h. The mixture was heated in an oil bath for 1 day at 100 °C. The pH of the solution is adjusted to 7	Wet chemical plus dialysis method	1–7	420/515	—	52 & (2018)
		Filtered by 0.45 μm PTFE membrane and purified by dialysis bag of 500–1000 Da for 3 days					
S, P-GQDs	CA + sodium phytate + anhydrous sodium sulfate	The mixture of CA, sodium phytate, and Na_2_SO_4_ was heated at 180 °C for 7 h	Hydrothermal treatment	3.5	340/440	15.6	55 & (2019)
		Centrifugation and dialysis of a prepared solution by using a dialysis bag (3500 Da) for 36 h					
P, S-GQDs	Graphite rod in a solution of sodium phytate, Na_2_S, and NaOH	Sodium phytate and Na_2_S were mixed in NaOH solution and used as electrolytes. The graphite rod as anode and Pt sheet as cathode dipped into prepared solution and voltage of 5 V was applied for 6 h	Electrolysis method	3.2	440/530	39.5	56 & (2019)
		Filtered by 220 nm filter and dialyzed by a dialysis bag (3500 Da) for 48 h					
B, N-GQDs	GO, boric acid, and ammonia solution	GO was dispersed in distilled water and sonicated for 10 min. Then, the addition of ammonia and boric acid. The mixed solution was heated 180 °C for 20 h	Hydrothermal treatment	3.8	322/422	5.1	54 & (2020)
		Filtered by 0.22 μm membrane and purified by a dialysis bag of 3000 Da for 2 days					
B, N-GQDs	CA + boric acid + urea	The mixture of CA, boric acid, and urea was heated for 2 h at 200 °C in an oven	Pyrolysis	2.0	357/464	17.1	51 & (2020)
		Centrifuged (10 000 rpm) many times and filtered by 0.22 μm microporous membrane. Further, dialyzed by using a dialysis bag (3000 Da) for 48 h					

We found from Tables 1 and 2 that different methods, precursors, and timing have been used for the synthesis of heteroatom doped GQDs. Table 3 represents the advantages and disadvantages of different methods used for the synthesis of heteroatom doped GQDs. Based on the information provided by Tables 1–3, we summarized all the methods based on their cost, efficiency, resources, timing, and safety. The synthesis based on microwave treatment was cheap, moderately efficient, very less time consuming, and safe. The synthesis based on the hydrothermal method was cost-effective, highly efficient, using economical resources, time-consuming, and safe. The wet chemical method based on acid treatment using coal was cheap, less efficient, a long-time process, but not safe. The electrochemical methods for the synthesis of heteroatom doped GQDs were highly efficient and safe, but expensive and time-consuming. According to the above literature, it was found that the hydrothermal method was most effective and mostly used for the synthesis of heteroatom doped GQDs.

**Table 3:** Advantages and disadvantages of different synthesis methods for heteroatom doped GQDs

Heteroatom doped GQDs	Method (precursors)	Advantages	Disadvantages	Ref. & (year)
N-GQDs	Hydrothermal (GO and ammonia)	- low cost	- Long synthesis process	30 & (2012)
		- Potential for large scale production		
		- No strong acid treatment		
	Multi-step hydrothermal (CA + hydrazine)	- Cheap	- Highly toxic precursor (hydrazine)	46 & (2014)
		- Highly crystalline – uniform particle size	- Multi-step method	
	Hydrothermal (CA + dicyandiamide)	- Excellent photostability	- Long purification time	47 & (2014)
		- Highly crystalline	- pH-sensitive	
		- Good QY		
		- One-pot synthesis process		
	Hydrothermal (CA + ammonia)	- Facile	None	19 & (2014)
		- Low cost		
		- Less time for synthesis and purification		
		- One-step synthesis process		
	High temperature treatment (CA + glycine)	- Simple	- pH-dependent PL intensity	25 & (2016)
		- Inexpensive		
		- Only 5 min synthesis process		
		- Highly soluble in water		
	Microwave irradiation (glucose + ammonia)	- Simple	- Low QY	24 & (2016)
		- Only 1 min synthesis process	- Less doping of nitrogen content	
		- Low cost		
	Electrochemical method (*o*-phenylene-diamine)	- High quantum yield	- Complex set-up	32 & (2016)
		- Large scale production	- Expensive	
	Hydrothermal (CA + ethylenediamine)	- High stability	- Large particle size	31 & (2017)
		- Low cost		
		- Easy synthesis		
	Chemical vapor deposition (chitosan)	- Cheap precursor	- Complex process	48 & (2018)
		- No use of harmful acids	- Specific conditions	
		- Single-step process		
	Chemical vapor deposition (C_60_/nitrogen plasma)	- One-step process	- Complex set-up	29 & (2018)
		- Uniform particle size	- Specific conditions	
	Electrochemical (N-CNT/N-graphene in ammonia solution)	- Highly stable	- Specific membrane for purification	49 & (2019)
		- Highly crystalline structure		
B-GQDs	Hydrothermal (boron-doped graphene)	- Homogenous and stable at room temperature	- Multi-step	50 & (2014)
		- Uniform particle-size	- Strong acid treatment	
			- Long synthesis process	
			- Long purification steps	
	Electrolyzing method (graphite rod in 0.1 M borax)	- Simple	- Specific purification conditions	34 & (2016)
		- Label-free		
		- Strong fluorescence		
	Electrochemical (boron-doped graphene rods)	- High stability	- Long synthesis process	33 & (2016)
		- Highly crystalline		
		- Good electrical activity		
	High-temperature treatment (VPBA and boric acid)	- Two-photon fluorescence	- Low quantum yield	37 & (2016)
		- NIR imaging	- Long purification process	
	Hydrothermal (1,3,6-trinitropyrene and borax)	- Easy synthesis	- Particular purification steps	35 & (2019)
		- High yield		
		- Bright fluorescence		
		- One-step process		
	Microwave irradiation (boron carbide)	- High photoactivity	- Multi-step process	38 & (2019)
			- High temperature	
			- Long purification process	
S-GQDs	Electrolyzing method (graphite in sodium *p*-toluene sulfonate aqueous solution)	- Good electrochemical reaction	- Filter membrane required	43 & (2014)
		- Good surface chemical reactivity		
		- High photostability		
	Hydrothermal (1,3,6-trinitropyrene + NaOH + Na_2_S)	- High yield	- Low quantum yield	41 & (2016)
		- Stable fluorescence within a wide range of pH	- Two dialysis bags used for purification	
		- High crystallinity		
		- Uniform size		
	Hydrothermal (MPA)	- One-pot synthesis	- Long purification process	42 & (2017)
		- Single-layer graphene structure		
		- Uniform size		
	Hydrothermal CA + powdered S	- Cheap and non-toxic precursor	None	44 & (2018)
		- Low-cost synthesis process		
		- High water solubility		
	Hydrothermal (sugarcane molasses)	- Green synthesis process	- Various grade Gooch crucibles are required for filtration	45 & (2018)
		- Good quantum yield		
		- Economical		
		- Facile		
P-GQDs	Electrochemical (sodium phytate)	- Good electrochemical reaction	- Long purification process	39 & (2017)
		- One-step synthesis process		
N, F, S-GQDs	Microwave treatment (MWCNTs in a customized ionic liquid medium)	- One-step synthesis	- Specific conditions for filtration	59 & (2015)
		- High quantum yield		
		- High yield		
N, S-GQDs	Hydrothermal (1,3,6-trinitropyrene, thiourea, DMF, and NaOH)	- High yield	None	53 & (2017)
		- Single-layer graphene structure		
		- Uniform size		
		- High crystallinity		
N, S-GQDs	Hydrothermal (CA + thiourea)	- Good quantum yield	None	18 & (2017)
		- One-pot synthesis		
		- Easy synthesis		
		- Cost effective		
B, N-GQDs	Solvothermal (*p*-phenylenediamine + formylphenylboronic acid)	- Facile	- Specific conditions for purification	60 & (2017)
		- One-pot synthesis method		
		- Cheap		
N, S GQDs	High temperature pyrolysis (CA + l-cysteine)	- High quantum yield	- Long purification time	61 & (2017)
		- Short synthesis process		
		- High ECL efficiency		
N, P, S-GQDs	Wet chemical plus dialysis method (anthracite coal)	- Cheap precursor	- Strong acid treatment	52 & (2018)
		- Facile oxidation process	- Harsh conditions for synthesis	
			- Long process for purification	
S, P-GQDs	Hydrothermal (CA, sodium phytate, and anhydrous sodium sulfate)	- High S and P doping	- Long purification time	55 & (2019)
		- High water solubility		
		- Good stability		
		- Facile synthesis		
P, S-GQDs	Electrolysis method (graphite rod in a solution of sodium phytate, Na_2_S, and NaOH)	- One-step electrolysis	- Long purification time	56 & (2019)
		- Good electrolysis reaction	- Complex set-up	
B, N-GQDs	Hydrothermal (GO, boric acid, and ammonia solution)	- One-pot synthesis	- Low quantum yield	54 & (2020)
		- Simple		
		- Cost-effective		
B, N-GQDs	Pyrolysis (CA, boric acid, and urea)	- Easy synthesis	- Specific conditions and long purification process	51 & (2020)
		- Cheap		
		- High crystalline		

## 3. Structure and characterizations of heteroatom doped GQDs

**Fig. 5:**
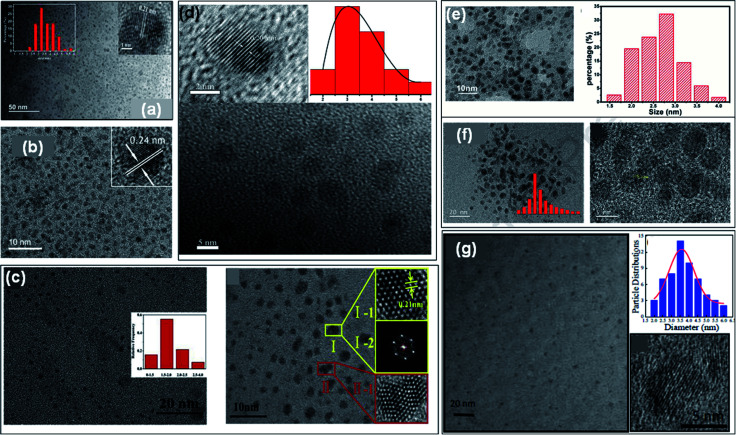
(a) TEM image and its HRTEM image (inset) of undoped GQDs,^13^ TEM and HRTEM images with particle size distribution histogram and lattice spacing of B-GQDs (b),^35^ N-GQDs (c),^62^ S-GQDs (d),^44^ P-GQDs (e),^39^ B, N-GQDs (f),^54^ and N, S-GQDs (g),^18^ respectively. Part (a), (b), (c), and (d) are reproduced from ref. 13, ref. 35, ref. 62, and ref. 44 with permission from [Elsevier], copyright [2018], [2019], [2020], and [2018], respectively. Part (e) is reproduced from ref. 39 with permission from [PCCP Owner Societies], copyright [2017], parts (f) and (g) are reproduced from ref. 54 and ref. 18 with permission from [Elsevier], copyright [2020] and [2017], respectively.

GQDs contain one, two, or more layers of graphene sheets having a thickness of less than 10 nm with a lateral size of 100 nm. The sizes of doped GQDs with different heteroatoms were ranged from 3 to 20 nm (Tables 1, and 2) and also consist of less than 5 layers of graphene sheets. The particle size distribution, shape, and surface morphology of heteroatom doped GQDs were investigated by transmission electron microscopy (TEM) images. The crystalline nature and height of GQDs were evaluated by high-resolution TEM (HRTEM) and atomic force microscopy (AFM) images, respectively. Fig. 5a represents a TEM image of undoped GQDs with narrow particle size distribution. HRTEM image (inset Fig. 5a) representing the crystal structure of undoped GQDs with 0.21 nm of lattice spacing that corresponds to the (100) crystal plane. The above result declared that there were crystal cores of graphitic sp^2^ carbon atoms present in the nanoparticles.^13^ Similarly, the TEM and HRTEM images with particle size distribution histogram of doped GQDs such as B-GQDs, N-GQDs, S-GQDs, P-GQDs, B, N-GQDs, and N, S-GQDs were represented in Fig. 5b, c, d, e, f and g, respectively. These TEM images displayed that the surface morphology of GQDs was mostly spherical and nanoparticles were mono-dispersed. The particle size of different GQDs was evaluated by the particle size distribution histogram. The HRTEM images of B-GQDs, N-GQDs, S-GQDs, B, N-GQDs, and N, S-GQDs showed a lattice spacing of 0.24 nm, 0.21 nm, 0.20 nm, 0.22 nm, and 0.21 nm, respectively. This result showed that heteroatom doped GQDs have (100) lattice planes corresponding to the graphite lattice structure.

**Fig. 6:**
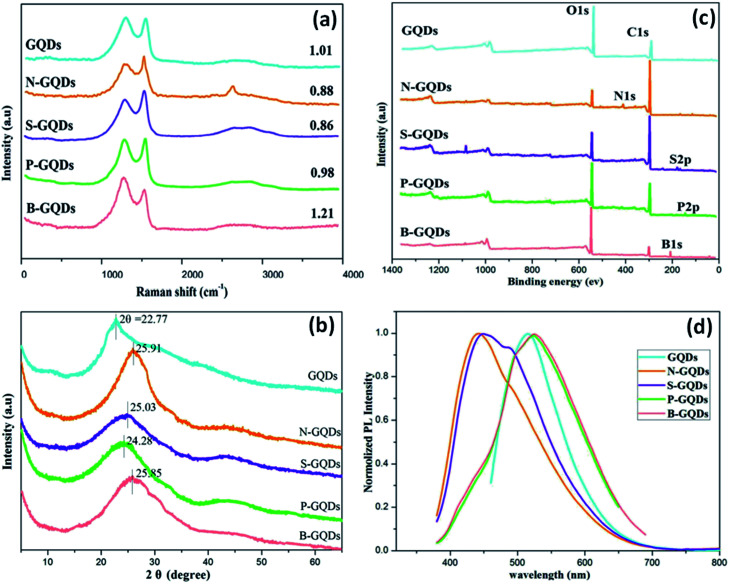
Raman spectra (a), XRD patterns (b), XPS spectra (c), and PL emission spectra excited at 360 nm (d) of GQDs, N-GQDs, S-GQDs, P-GQDs, and B-GQDs, respectively.^17^ Part (a), (b), (c), and (d) are reproduced from ref. 17 with permission from [Royal Society of Chemistry], copyright [2016].

Raman spectroscopy is used to examine the degree of disorder in the structure of the carbon matrix based on the different kinds of defects, for example, grain boundaries, point defects, stress, strain, stacking faults, edges, doping, vacancies, functional groups, and many others.^45^ The Raman spectra of GQDs and heteroatom doped GQDs consist of two bands were observed *i.e.*, G band and D band. G band represents the E_2g_ vibration modes of the aromatic sp^2^ carbon domains and the G band corresponds to the defects and structural disorder of sp^2^ domains.^38^ Mostly, the intensity fraction of D to G band (*I*_D_/*I*_G_) is utilized to investigate the order of structure among crystalline and amorphous systems of the graphitic domain. The values of *I*_D_/*I*_G_ depend upon the type of method used for the synthesis of GQDs. A high value of *I*_D_/*I*_G_ of heteroatom doped GQDs showed many defects on the surface of GQDs. Wang *et al.*^17^ determined the *I*_D_/*I*_G_ value for GQDs, N-GQDs, S-GQDs, P-GQDs, and B-GQDs which were 1.01, 0.88, 0.86, 0.98, and 1.21, respectively (Fig. 6a). The number of defects follows the order such as B-GQDs > GQDs > P-GQDs > N-GQDs > S-GQDs.

Mostly, X-ray diffraction (XRD) patterns of undoped and heteroatom doped GQDs show a broad peak corresponds to the (002) graphitic lattice plane. The broad peak in the XRD pattern is due to the reduced size of the quantum dots. The small size of GQDs is responsible for its broad peak in the XRD pattern.^63^ The crystalline or amorphous nature of GQDs was investigated by the XRD pattern. Doping of heteroatoms such as boron having a greater size and less electronegativity than carbon can increase the interlayer spacing. In B-doped GQDs, the peak gets shifted towards the lower angle due to the participation of the boron atom in its graphitic structure which results in enlarging the interlayer spacing.^8^ In the case of N-doped GQDs, no increase in interlayer spacing was shown due to the less atomic radius of a nitrogen atom (65 pm) as compared to a carbon atom (70 pm). But the electronic structure gets altered due to the lone pair of the nitrogen atom. Therefore, in the case of N-GQDs, the peak shift to a higher angle corresponds to π–π stacking of graphene along with structural disorder due to doping of nitrogen. Fig. 6b shows the XRD pattern of GQDs, N-GQDs, S-GQDs, P-GQDs, and B-GQDs in which graphitic peak (002) was located at 22.77, 25.91, 25.03, 24.28 and 25.85°, representing interlayer spacing of 0.391, 0.344, 0.356, 0.367 and 0.345 nm, respectively. It was reported that, the interlayer spacing of undoped GQDs is greater as compared to graphite (0.334 nm) due to the existence of oxygen-containing groups.

The presence of chemical groups and heteroatom (N/S/P/B) as dopants slightly increases the interlayer spacing from graphite. But the large compacted interlayer spacing in heteroatom doped GQDs may be due to pyrolysis at high temperature.^17^ The functional groups (such as hydroxyl, carbonyl, epoxy/ether, and carboxylic acid groups) and elemental composition of heteroatom doped GQDs are determined by Fourier transform infrared (FTIR) and X-ray photoelectron spectroscopy (XPS) spectra, respectively.^64^ The electrochemical and optical properties depend upon the synthesis methods, heteroatoms, functional groups, and surface defects. Wang *et al.*^17^ determined the XPS measurements of undoped GQDs and heteroatom doped GQDs in which a major peak of graphitic carbon C 1s at 284.0 eV and O 1s at 531.2 eV was found. Other than C 1s and O 1s, the N 1s at 399.1 eV, S 2p at 164.3, P 2p at 133.8 eV, and B 1s at 192.4 eV have appeared in the XPS spectra of N-doped GQDs, S-doped GQDs, P-doped GQDs, and B-doped GQDs, respectively (Fig. 6c). XPS spectra confirmed the doping of heteroatom N/S/P/B into the framework of GQDs. They determined that the atomic ratio of different heteroatoms *i.e.*, N, S, P, and B with respect to C was 17.36%, 1.60%, 1.78%, and 40.8%, respectively. Due to higher atomic radius of S12 and P, the atomic ratio of S and P was lower than N-GQDs and B-GQDs. Also, the intensity of O 1s increases due to doping of B as well P by bond formation with O and elimination of O by heat treatment cannot be achieved.

## 4. Properties of heteroatom doped GQDs

Properties of GQDs like optical properties are highly dependent on the synthesis method and precursors.^31^ Heteroatom doping provides a route for changing the optoelectronic properties of materials consists of graphene.^65^ The intrinsic properties of GQDs such as electronic structures, chemical activity, quantum yield, and band gap can be tuned by its heteroatom doping.^66^

### 4.1. Optical properties

Doping with heteroatoms (like N, S, B, P, and F) is an active method to vary the optical properties of the GQDs, and doping with N, S, F on GQDs can lead to open up a small bandgap in its electronic structure.^59,67^ The heteroatom doping on the GQDs manipulate its electronic structure, chemical structure, and surface defects state that result in variation of optical properties.^68^ PL spectra give information about the number of photons emitted whereas the absorption spectra exhibit light absorption.

#### 4.1.1 Absorption

**Fig. 7:**
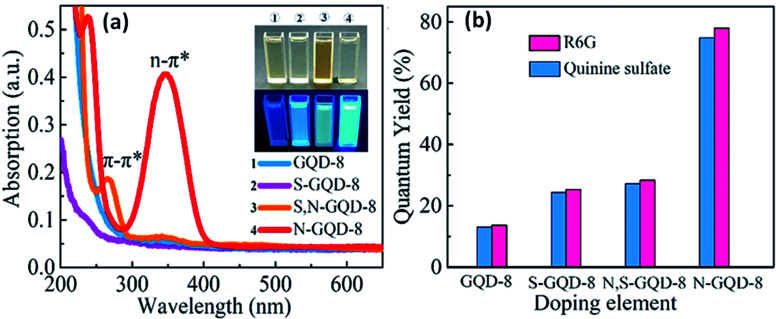
PL spectra (a) and quantum yields (b) of heteroatom doped GQDs *i.e.*, GQDs, S-GQDs, N-GQDs, and also S, N-GQDs.^70^ Part (a) and (b) are reproduced from ref. 70 with permission from [Elsevier], copyright [2019].

Heteroatom-doping increases the quantum yield of GQDs by reducing the energy difference among low energy excited states which further enhances the radiative transitions probability. The shift in the absorption spectra of doped GQDs depends upon the hybridization of outmost orbitals among edge heteroatom-doping and GQDs.^69^Fig. 7a represents the UV spectra of four kinds of GQDs *i.e.*, GQDs, S-GQDs, N-GQDs, as well as S, N-GQDs in which dual absorption bands were mainly examined in the case of S, N-GQDs, and N-GQDs. The absorption peak at 240–270 nm was due to π → π* transition of the sp^2^ domain and the intense peak at 350 nm was due to n → π* transition. The inset image of Fig. 7a shows the picture of all GQDs under UV and visible light irradiations in which all GQDs representing blue emission.^70^ The quantum yield of GQDs efficiently improved by doping of heteroatoms as shown in Fig. 7b. N-GQDs showed the greatest value of quantum yield *i.e.*, 78% in reference with R6G whereas S, N-GQDs, S-GQDs, and undoped GQDs showed 32%, 28%, and 14%, respectively. The variation in the transition energy levels by heteroatom doping in GQDs can be determined by absorption spectra. Yang *et al.*^71^ compared the absorption spectra of undoped GQDs (U-GQDs), N-GQDs, as well as B-GQDs. They found that N-GQDs showed a blue shift due to electron-rich enrichment in GQDs by an electron-rich nitrogen atom, whereas B-GQDs showed a red-shift due to electron deficiency in GQDs caused by electron-deficient boron atom. They also found that the intensity of the band also decreases mutually for N-GQDs and B-GQDs due to a decrease in the delocalized electrons in the π–π* transitions in the core.

Feng *et al.*^66^ determined the effect of four heteroatoms including B, N, P, and S along with 3 different patterns on GQDs through time-dependent density functional theory (TD-DFT). They investigated the UV-visible spectra and HOMO–LUMO gaps for determining the relationships among the optical properties and doping of heteroatom on GQDs. They found that the heteroatoms make the formation of hybridization and therefore, a greater orbital hybridization leads to a high decrease in the HOMO–LUMO gap. In a comparison study, they found that the absorption spectra of N and S doped GQDs showed a minor hypsochromic shift, while B and P doped GQDs showed a blue shift for edge-doped GQDs in the pentatomic ring. Liu *et al.*^58^ observed the absorption spectra of N, P-GQDs in which dual absorption peaks located at 275 nm and 350 nm corresponds to π–π* transitions of aromatic π-domain in the carbon core and the absorption peak at 350 nm corresponds to n–π* transition of C=O, C=P, and C=N bonds, respectively.

#### 4.1.2 Photoluminescence (PL)

The origin of PL is depend on various models such as QCE, surface functional groups, zig–zag edge states, the electronegativity of heteroatoms, and the huge red-edge effect.^16^ GQDs with different emissive color were synthesized by various methods till now, but their PL mechanism is under debate. There are two main categories of emission, one is intrinsic recombination which is related to band-gap (carbon-core) and the second one is extrinsic recombination which is related to the surface.^72^ In the case of GQDs consists of a smaller number of surface functional groups, the bandgap of carbon core is the main dominating factor for the origin of PL. The quantum confinement effect (QCE) occurs when quantum dots have a smaller size than the exciton Bohr radius.^73^ Xie *et al.*^74^ found that the size of GQDs reduces with the increase in hydrothermal temperature and the blue shift was observed as their size decreases, which confirm that the PL emission is dependent upon size. The optical properties get changed with the variation in the energy levels by doping of heteroatoms on GQDs.^75^ The doping of heteroatom in the structure of GQDs modulates their electrical properties by decreasing band gap and enhancing optical absorption.^57^ Therefore, it desires to study the emissive states present in doped GQDs and the role of doped heteroatoms in the emissive mechanism.^71^

The GQDs consist of different surface functional groups and sp^2^ & sp^3^ carbon atoms which induce the surface state defects. The PL intensity decreases or increases with the variation of pH due to protonation and deprotonation. The reduction or oxidation of functional groups like –COOH and –OH present on the surface of GQDs put an impact on the PL intensity.^72^ This confirm that the optical properties of the GQDs are dependent upon the surface functional groups. The chemical modification of GQDs with different functional groups results in surface defects and put an impact on the PL intensity.^76^ Rajender *et al.*^77^ proved the PL emission of GQDs depends upon the surface defects by measuring the PL intensity in various solvents with different dielectric constants. They found that, the shift in the PL emission of GQDs present in different solvents was due to surface defects. The surface passivation with organic reagents has some drawbacks, like cytotoxic whereas doping with metal atoms results in non-uniform doping due to larger size than C atoms. Doping with non-metal atoms such N, S, P, B also induces the surface defects with uniform doping and enhances the fluorescence quantum yield of GQDs.^78^ Feng *et al.*^66^ found that the heteroatom doping assists charge transfer to occur in the excited state and diminish the electron and hole separation in the graphene structure. They found that, the big atoms incorporated on the basal plane show a crucial part in the recombination of electron density distribution in heteroatom doped GQDs by altering the geometry configuration. Also, the large atoms enlarge the HOMO–LUMO gap by transforming the sp^2^ hybridized carbon atom to sp^3^ hybridized. Various studies have been reported for the N-GQDs in which fluorescence is enhanced by doping of N atoms. The N atoms doped on the GQDs act as electron-rich active sites and enhance the radiative recombination and decrease non-radiative recombination.^18^ Also the sensing activity of N-GQDs depends upon the coordination performance of graphitic N atoms.^32^ Wang *et al.*^17^ observed that the emission wavelengths of GQDs (513.8 nm), N-GQDs (443.6 nm), P-GQDs (526.8 nm), and B-GQDs (525.2 nm) excited at 360 nm (Fig. 6d). In comparison with GQDs, N-GQDs and S-GQDs show a blue shift in the emission wavelength whereas P-GQDs and B-GQDs display a red shift in emission wavelength. They found that the emission wavelength follows the order *i.e.*, B-GQDs > P-GQDs > GQDs > S-GQDs > N-GQDs.

In the case of some heteroatom doped GQDs, the synergistic effect of both QCE and surface defects leads to the origin of PL. Wang *et al.*^55^ prepared S, P-GQDs with large ratios of heteroatom doping *i.e.*, S (9.66 at%) and P (3.34 at%). They found that the S, P-GQDs show high quantum yield as compared to monoatomic doped GQDs. The electronegativity variance among P (2.19) and S (2.58) result in the easy release of valence electrons of P as compared to S and also the high electron density of oxygen on GQDs enhances their synergistic effect. PL behavior of S, N-GQDs depends upon both the carbon core and surface functional groups.^79^

The PL mechanism is also based on the free zig–zag sites. Pan *et al.*^80^ prepared GQDs by hydrothermal cutting of GO and they obtained blue-emissive GQDs based on the free zig–zag sites effect. Radovic *et al.*^81^ revealed that zig–zag edges act like carbene sites (triplet state) and armchair sites act like carbyne sites (singlet state), both result in the origin of PL in GQDs. Lingam *et al.*^82^ studied the role of zig–zag edge sites in the origin of PL of GQDs with an experimental prove. They compared the PL results of GQDs, graphene nanoribbons (GNR), and carbon nano-onions (CNOs) and observed that the CNOs did not show any PL signal due to having a cage-like structure (no free edges). They found that, the free zig–zag edges present in GQDs are responsible for the PL emission. Yang *et al.*^83^ found that the free zig–zag sites of GQDs get protonated and showed less PL emission due to breakage of the emissive triplet state. On the other hand, the free zig–zag sites get restored in alkaline solution, result in PL recovery. Kumar *et al.*^84^ prepared amino-functionalized GQDs and found that the origin of PL emission depends upon the zig–zag effect. The PL of GQDs is also pH-dependent, such that on switching the pH between 1 and 12, the PL properties vary significantly. This process of varying pH is highly ascribed to the protonation and deprotonation of the free zig–zag or transmission sites of GQDs under alkaline and acidic conditions, thereby affecting the PL wavelength.^85,86^

The excitation-dependent and excitation-independent PL behavior of GQDs are still in debate. In the literature, different mechanisms were reported for both excitation-dependent and independent PL emission of GQDs. The excitation-dependent PL emission can be tuned by varying its excitation wavelength only and no need to modulate the structure of GQDs. Thus, this can be useful in various applications. The deviation in the energy band gap due to the size distribution of GQDs, result in excitation-dependent PL emission correlated with the QCE. But, excitation-independent PL emission was found in the case of large size distributions of GQDs. This proves that, size is not only the factor for the excitation-dependent PL emission.^87^ Among the different sizes, shapes, or surfaces of GQDs developed by different methods, some of them still have the same excitation-dependent PL properties.^88^ The excitation-dependent PL properties of GQDs are due to their different sizes, surface defects, QCE, conjugated sp^2^ π-domains, and zig–zag edge sites.^88^ Also, the functional groups present on the edges of GQDs introduce many excited states which result in radiative recombination of excited electrons and further lead to PL emission at different wavelengths.^52^ The surface defects also responsible for the excitation-dependent PL emission in GQDs. But there is no united agreement for surface state defects on the excitation-dependent PL emission due to having non-radiative traps.^89^ Heteroatom doping of GQDs also affects the size and surface functionalization of GQDs, which put an impact on its PL properties. Masteri-Farahani *et al.*^90^ found that the change in the size of S, N-GQDs leads to produce distinct sp^2^ clusters, and the electronic transitions from these sp^2^ domains resulted in PL emission shift. Also, the different functional groups present at the edges of S, N-GQDs lead to a generation of emissive traps between HOMO and LUMO levels due to which Stoke shift was observed along with excitation.

In the excitation-independent, the sample shows the same PL emission spectra at different excitation wavelengths. Wang *et al.*^91^ determined that S, N-GQDs showed excitation-independent PL and blue emission. They found that the excitation-independent PL was due to the uniform particle size of S, N-GQDs. Also, the doping of S eliminates the O-containing groups and increases the N-containing groups from the surface of GQDs *via* a cooperative effect that results in a new type of surface defect state. Zhu *et al.*^92^ observed that the GQDs showed excitation-independent PL behavior depends upon the surface states. They observed from the TEM images that, the particle size was not highly uniform. Thus, surface defects were more dominant for the excitation-independent PL emission as compared to the different particle sizes. Gu *et al.*^93^ prepared GQDs exhibiting excitation-independent PL emission due to more dominating surface state defects rather than size. The surface states consist sp^2^ carbon domain which is more uniform. Dong *et al.*^94^ observed excitation-independent PL emission of GQDs due to uniform distribution of both particle size and the surface defects consists of sp^2^ domains. Zheng *et al.*^29^ found that the N-GQDs were incorporated in the carbon framework having uniform size and result in excitation-independent PL emission.

### 4.2. Electrochemiluminescence (ECL) properties

ECL is built on the emission of light which includes the electron transfer reaction of high-energy species produced at electrodes, which get transferred to an excited state and there will be redox induced emission of light.^61^ ECL consists of electrochemistry with chemiluminescence and applied in various fields such as environmental challenges, medical, pharmaceutical, *etc.*^95^ There is no need for any light sources in ECL, and also the time & position of ECL emission is measured by varying an electrode potential. This enhances the selectivity of ECL-based systems which results in their great advantages in bioanalysis. There are various materials used as ECL materials such as luminal^96^ and ruthenium^97^ compounds. But these materials have limitations due to their toxic nature for organisms. In particular, heteroatom doped GQDs have been used as an effective luminescent material in the ECL sensors due to their less cytotoxic nature and exceptional biocompatibility as compared to traditional ECL materials.^33^ The GQDs have both excellent electrochemical properties as well as outstanding luminescence properties.^56^ The minor graphene fragments in GQDs enhances transfer of electron in all 3 spatial dimensions. Large surface area, great charge transport, excellent mechanical flexible nature, and good thermal stability are due to the QCE and edge effect of GQDs that enhance its ECL properties. Heteroatom doping on the GQDs enhances their optical and chemical properties. The heteroatoms doped GQDs were used further as ECL emitters for the determination of small biomolecules, proteins as well as organic compounds. The pristine GQDs lacking passivation showed low quantum yield and a large value of electro-oxidation potential, resulting in its limited application for practical uses. Therefore, heteroatom doping of GQDs can advance their ECL properties by varying their charge density distribution and band gap.^56^ For example, N doped GQDs enhance active sites on the surface of GQDs that make them supreme luminescence material and capable catalysts. These properties of N-GQDs result in the preparation of highly efficient ECL sensors by combining both luminescent and electrochemical methods.^98^ Peng *et al.*^56^ determined that the P, S-GQDs having yellow-green PL emission showed good ECL performance in the existence of K_2_S_2_O_8_. Zhang *et al.*^61^ observed that the ECL N, S co-doped GQDs in the existence of K_2_S_2_S_8_ which act as a co-reactant showed ECL efficiency (32%) as compared to Ru(bpy)_3_Cl_2_/K_2_S_2_O_8_ and also 5.8 fold increased by a system with undoped GQDs.

### 4.3. Electrochemical properties

GQDs have unique electrochemical properties which result in a large number of electrochemical applications.^99^ For example, it was reported that B and N doped GQDs showed great specific capacity in lithium and sodium ions batteries as compared to undoped GQDs. This was due to the presence of electron-deficient (B) and electron-rich (N) heteroatom as a dopant in carbon lattice, which increases the absorption and storage capacity of Li ions.^8^ GQDs showed brilliant photophysical and electrochemical properties to prepare cheap, less toxic, high biocompatible sensors.^100^ The variation in the sizes of GQDs can put a great impact on their electronic properties GQDs. The heteroatom doping greatly enhances their electrochemical activity by improving the surface area, surface defects, solubility, and the number of active sites.^101^ Doping of GQDs with heteroatoms can effectively tune its band gap due to which its electrochemical properties get modulated.^102^ Nowadays, heteroatom doped GQDs were considered as a novel electrocatalyst in the electrochemical reaction. It was found by theoretical calculations that the synergistic coupling effects among the heteroatoms changed the electronic structure of carbon. This results in the activation of strong absorption or desorption active sites present on the surface of GQDs for enhancing electronic transport.^103^ Though, GQDs have not good conductivity. The electrical conductivity of GQDs enhances with the heteroatom doping by tuning its intrinsic properties.^104^ Heteroatom doping improves the electronic performance of GQDs by increasing the number of actives sites.^105^ N-Doped GQDs enhanced the defects in the aromatic lattice structure of GQDs. Heteroatom doped GQDs improved the electrochemical performance of hybrid materials. For example, N-GQDs were acting as a reducing agent and also as a support for the preparation of Au nanoparticles. In the Au NP@N-GQDs hybrid, N-GQDs enhance the electrochemical activity of the hybrid by providing a great specific surface area, and good stability.^106^ It was reported that the co-doped GQDs can also successfully provide more active sites for electrocatalytic activity as compared to single heteroatom doped GQDs. This was because of the synergistic coupling effect among various heteroatoms. The edge effect and B, N-doping of GQDs, and great electrical conductivity of graphene in B, N-GQDs/graphene hybrid leads to provide significant electrocatalytic activity.^107^

## 5. Sensing application

Metal ions serve massive roles in various physiological and pathological pathways.^108^ For instance, Fe^3+^ plays a crucial role such as enzymatic catalysis, cellular metabolism, nucleic acid (DNA and RNA) synthesis owing to its incorporation in the structure of proteins and enzymes.^109^ Similarly, Cu^2+^, act as a cofactor for many enzymes and play a critical role in physiological functions. But the abnormal levels of these heavy metal ions (Hg^2+^, Ag^+^, Cu^2+^, Fe^3+^, and Pb^2+^) in the body cause multiple disorders and health issues.^110^ Besides these metal ions, small organic molecules, proteins, and macromolecules also play very important roles in the biological, medical, and environmental systems.^111–113^ There are various insecticides or pesticides *etc.* which affect the environment and their detection is needed.^114^ The excess or deficiency of these metal ions and biomolecules in the body leads to anemia, cancer, limiting oxygen delivery to cells, decreased immunity, organ dysfunction, and many more.^115^ This ultimately leads to significant demand for selective and efficient detection for early identification, quantification as well as early diagnosis of these diseases.

Nanomaterials are acting as promising probes for environmental safety.^116^ The interactions among GQDs and specific substances can originate diverse effects in the fluorescent intensity of GQDs. Due to their intrinsic fluorescent properties, the intensity of GQDs either gets reduced or enhanced.^117^ Based on this principle, a variety of sensors have been developed for the determination of different metal ions, macromolecules (like DNA, RNA, proteins), small organic molecules, insecticides, and pesticides with good selectivity and sensitivity.^108^ Nowadays, GQDs-based sensors and biosensors are rapidly developed with stable PL, unique ECL, and electrochemical properties that are extremely sensitive for small perturbations.^118^ Therefore, GQDs-based sensors can be utilized for the selective detection of metal ions and biomolecules.^119^ The mechanism of these sensors is mostly based on the occurrence of charge transfer between the metal ion and GQDs due to shorter distance among them, thereby resulted in the quenched or enhanced PL. The functional groups present at the edge of GQDs enhance its water solubility as well as provides the potential for functionalization with many inorganic, organic, polymeric, and biomolecules. The ECL and electrochemical properties of GQDs are because of the electron-donating and accepting tendency of the GQDs owing to high surface area and large number of edge sites.^86,118^ These attractive properties of GQDs lead to enhance their application in biosensing.

In recent years, the GQDs-based sensors have been extensively used in the sensing probe for the efficient sensing of metal ions and biomolecules as compared to other standard methods. But the detection sensitivity decreases due to the poor quantum yield of GQDs. Therefore, to improve the performance of GQDs, they have been functionalized by surface modifications. And, this can be achieved by controllable oxidation degree, surface functionalization, and heteroatom doping with heteroatoms (N, S, P, and B) to enhance the quantum yield of GQDs.^117^ The doping and co-doping of GQDs generates additional coordination sites and provides more defects in the structure of prepared GQDs which eventually improves the physical and chemical properties, for example, chemical reactivity, optical properties, and electronic framework of GQDs by tuning their intrinsic properties.^85,120^ These heteroatom doped GQDs based biosensors have thus provided a new platform for early diagnosis of several diseases, improved biological agents, bioenvironmental monitoring, and safety assessment. Therefore, these GQDs become suitable probes for various sensing applications. The distinctive and recently designed GQDs-based biosensors are listed in Table 4.

**Table 4:** The emerging sensors based on heteroatom doped GQDs

Sensor	Materials	Detection target	Detection range	LOD	Advantages	Disadvantages	Ref. & (year)
PL sensor	Phosphorylated peptide–graphene quantum dot (GQD)	Protein kinase	1.1–1.0 unit mL^−1^	0.03 unit mL^−1^	- Simple	- Less sensitive	136 & (2013)
					- Fast detection		
					- Drug development		
	B-GQDs	Glucose	0.05–10 mM	0.01 mM	- Label free sensing	- Sensitivity in mM range	50 & (2014)
					- High selectivity	- pH dependent detection	
	GQDs@GSH	Cu^2+^	0.1–1.0 μM	53 nM	- Act as dual probe	- Specific pH detection	137 & (2015)
					- Cell imaging		
					- High sensitivity		
	N-GQDs	Thia-cloprid	0.1–10 mg L^−1^	0.03 mg L^−1^	- Test strip-based sensor	- Long preparation process of test strip	123 & (2018)
					- High selectivity		
					- Portable sensor		
	GQD-polyacrylonitrile (PAN) membrane	Chlorine	10–600 μM	2 μM	- High reproducibility	None	118 & (2018)
					- No need of incubation time		
					- High stability of sensor		
					- Real sample detection		
	S-GQDs-Al_3_ + (S-single layered)	Phosphate ion (PO_4_^3−^)	0.25–7.5 μM	0.1 μM	- Label free	None	138 & (2018)
					- High selectivity		
					- High reproducibility		
					- Low cytotoxicity		
					- Detection in water sample		
	S, N-GQDs	Ascorbic acid	10–500 μM	1.2 μM	- Simple	- Less sensitive	79 & (2019)
					- Less time consuming	- No selectivity studies	
	S, N-GQDs	Ag^+^	12–125 μM	12.90 μM	- High selectivity	- pH dependent sensing	119 & (2019)
		Hg^2+^	12–125 μM	9.14 μM	- Real sample detection		
					- Wide range of detection		
	Maleimide-GQDs	Bithiol (GSH + Cys)	5–400 nM	1.69 nM	- Highly sensitive	- Long incubation time	139 & (2020)
					- Real sample detection		
					- High reproducibility		
	N-GQDs	Homo-cysteine	5 × 10^−11^ to 5 × 10^−8^ M	5 × 10^−11^ M	- Very low detection limit	None	111 & (2020)
					- PL sensing in living cells		
					- Excellent selectivity		
	N-GQDs	Hg^2+^	0.5–110 nM	0.08 nM	- High sensitivity	None	115 & (2020)
					- Wide detection range		
					- Real sample detection		
					- Simple		
	N-GQDs	Fe^3+^	0–200 μM	0.87 μM	- Good selectivity	- Less sensitive	135 & (2019)
					- Easy		
					- Simple		
					- Low cytotoxicity		
ECL sensor	MoS_2_-GQDs	2-Methyl-4-chlorophenoxyacetic acid	10 pM to 0.1 μM	5.5 pM	- Very sensitive	- Complex	127 & (2017)
					- Real sample sensing	- Expensive	
					- Good reproducibility		
	Au@Ag/GQDs	HULC (highly up-regulated in liver cancer)	1 fM to 5 nM	0.3 fM	- Ultrahigh sensitive	- ECL response dependent on thickness of Au@Ag nanoparticles	140 & (2018)
					- Good selectivity	- Long time for fabrication	
					- Good stability	- Complex	
	AuNP-GQDs	Carcinoembryonic antigen	0.1 pg mL^−1^ to 10 ng mL^−1^	3.78 fg mL^−1^	- High sensitivity	- Specific pH detection	141 & (2018)
					- High reproducibility	- Long fabrication process	
					- Good selectivity		
	GQDs-AuNPs	Glucose	0.1–5000 μM	64 nM	- Sensing in complex human serum	-Temperature dependent	125 & (2019)
					- Good selectivity		
	GQD/TiO_2_NTs	Prostate protein antigen	1.0 fg mL^−1^ to 10 pg mL^−1^	1 fg mL^−1^	- High stability	- Complex fabrication	142 & (2019)
					- Repeatable		
					- Biological sample sensing		
	NGQDs/boron nitride quantum dots	Folic acid	1.0 × 10^−11^ M to 1.0 × 10^−4^ M	5.13–10^−12^ M	- Very low detection limit	None	124 & (2019)
					- Good selectivity		
					- High recovery		
					- Simple		
					-Real sample sensing		
	AuNCs-GQDs	Pentoxifylline	7.0 × 10^−7^ to 1.2 × 10^−4^ mol L^−1^	9.0 × 10^−8^ mol L^−1^	- Good selectivity	None	95 & (2019)
					- Good reproducibility		
					- Easy fabrication		
	AuNP-GQDs-DNA S3 and DNA S2	Hg^2+^	0.01 nM to 100 nM	2.48 pM	- Ultrahigh selectivity	- Long incubation time	143 & (2019)
					- Excellent selectivity		
					- Real sample sensing		
	PICA/F–Au-GQDs [poly (indole-6-carboxylic acid)/flower–gold]	Aflatoxin B1	0.01–100 ng mL^−1^	0.00375 ng mL^−1^	- Very detection limit	- Long fabrication process	144 & (2020)
					- Wide detection range	- Specific temperature detection	
					- Real sample analysis		
	Ru(pby)_3_^2+^@N-GQDs	Ethosuximide (ESM)	5.00 × 10^−7^ to 1.00 × 10^−4^ mol L^−1^	3.00 × 10^−7^ mol L^−1^	- High selectivity	- Ratio dependent of composite	126 & (2020)
					- Real sample sensing	- Specific pH range for detection	
					- Good recovery		
	P, S-GQD	Okadaic acid (OA)	0.01–20 ng mL^−1^	0.005 ng mL^−1^	- Low detection limit	- Specific temperature and time for incubation	56 & (2020)
					- Low matrix effect		
					- Advance fabrication		
	N-GQDs	Quercetin	2.0 × 10^−9^ to 1.6 × 10^−6^ M	8.2 × 10^−10^ M	- Highly sensitive	- Complex	145 & (2019)
					- High accuracy		
					- Wide detection range		
					- High selectivity		
	GQDs/GNPs/GCE	Luteolin	1 × 10^−8^ to 1 × 10^−5^ M	1.0 nM	- Low detection limit	None	130 & (2019)
					- Good selectivity		
					- Real sample detection		
	GQDs-MWCNTs	DA	0.005–100.0 μM	0.87 nM	- Excellent selectivity	None	146 & (2020)
					- Real sample detection		
					- High sensitivity		
	GQDs@MWCNTs/GCE	Dopamine (DA)	0.25–250 μM	95 nM and 110 nM	- High sensitivity	- Time consuming	147 & (2020)
					- Wide range of detection		
					- Excellent electrocatalytic	- Expensive	
					- Real sample analysis		
	GC/GQDs-NF (NF-naflon)	Cd^2+^	20–200 μg L^−1^	11.30 μg L^−1^	- Real sample detection	- Specific conditions	120 & (2020)
					- High accuracy	- Less sensitive	
	GC/GQDs-NF	Pb^2+^	20–200 μg L^−1^	8.49 μg L^−1^	- Real sample detection	- Specific conditions	120 & (2020)
					- High accuracy	- Less sensitive	
	Au@Cu-MOF/N-GQDs	Patulin	0.001–70.0 ng mL^−1^	0.0007 ng mL^−1^	- Good selectivity	- Long fabrication process	129 & (2020)
					- Good accuracy		
					- Very low detection limit		
	MIP/GQDs-Pt nanoparticles	Sulfadimidine (SM_2_)	0.1 nM to 0.1 mM	0.023 nM	- Highly sensitive	- Particular conditions of fabrication	134 & (2020)
					- Computer simulation		
					- Real sample analysis		
	CdS/Au/GQDs	DA	0.1 to 350 μM	0.0078 μM	- Limit of detection lower than DA in biological tissues	- Complex process	133 & (2020)
					- Wide range of detection		

### 5.1. PL sensor

**Fig. 8:**
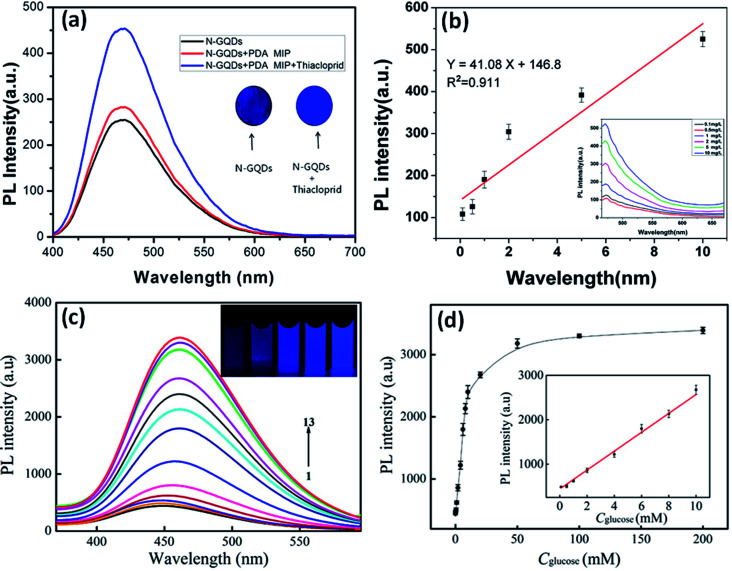
(a) PL spectra and the inset image of test strip of N-GQDs/N-GQDs with thiacloprid under the UV lamp,^123^ (b) standard curve between PL intensity and concentration of thiacloprid,^123^ (c) PL responses of B doped GQDs with increase in the glucose concentration (1 to 200 mM) and inset image shows increase in PL under UV lamp with increase in the glucose concentration from left to right (0, 2, 8, 20, and 50 mM),^50^ and (d) standard plot between PL intensity and concentration of glucose in phosphate buffer of pH value of 7.4.^50^ Part (a), (b) are reproduced from ref. 123 with permission from [Elsevier], copyright [2018], and part (c), (d) are reproduced from ref. 50 with permission from [American Chemical Society], copyright [2014].

GQDs exhibit excellent luminescence properties such as good photostability, high quantum yields, stable light-emitting, and biocompatibility which are mainly due to the QCE and the edge effect.^121^ The unique PL properties of GQDs make them capable sensors for the detection of biologically related ions, proteins, macromolecules, and metal ions based on PL turn-on/turn-off mechanisms.^122^ GQDs have shown variation in PL because of the changes in different parameters like concentration, size, pH, solvent polarity, excitation wavelength, heteroatom doping, surface functionalization, and many others. Selectivity is the crucial parameter for sensing applications. Therefore, various doped or functionalized GQDs-based PL probes were utilized for the efficient sensing of metal ions, small organic molecules, and biomaterials with ultra-selectivity and high sensitivity. Based on PL properties, Liu *et al.*^123^ prepared a test-strip based on fluorescence sensor by using MIP for thiacloprid sensing (Fig. 8a). The established polydopamine (PDA) MIP can specifically capture and selectively recognize the target thiacloprid. The thiacloprid which gets captured by the system can increase the PL intensity of N-GQDs effectively. A good relationship was found between the PL intensity and the different concentrations of thiacloprid (0.1–10 mg L^−1^) by N-GQDs/MIP sensor (Fig. 8b). Most importantly, this sensor provided a rapid, portable, and reliable analysis method for pesticide detection. Du *et al.*^115^ synthesized N-GQDs as the luminescent agent and GSH as the masking agent to detect Hg^2+^ in tap water. The addition of Hg^2+^ significantly reduced the PL intensity of N-GQDs, which was attributed to coordination reaction inducing the aggregation of N-GQDs. The prepared PL N-GQDs/GSH system showed high selectivity and sensitivity for the detection of Hg^2+^.

Zhang *et al.*^50^ have synthesized B-GQDs by using a hydrothermal approach for ultra-selective detection and sensing of glucose. The PL intensity of B-GQDs solution increases in the presence of different concentrations of glucose (1–200 mM) (Fig. 8c). Fig. 8d represents the plot of the PL intensity of B-GQDs with the different concentrations of glucose (1–200 mM) and the inset image reveals a linear curve for the determination of glucose (0.1–10 mM) with a limit of detection (LOD) value *i.e.*, 0.03 mM. They observed the enhancement in the PL intensity of B-GQDs due to limitations on the intermolecular rotations started by unusual interactions of B-GQDs and glucose. This was due to the reaction between the dual cis-diol components present in glucose and dual boronic acid groups located on the surface of B-GQDs. Therefore, B-doping on the GQDs provides a novel type of surface state that gives rise to a new PL enhancement mechanism after the interaction with glucose.^50^

### 5.2. ECL sensors

ECL is a powerful combination of chemiluminescence and electrochemistry which results in electrogenerated chemiluminescence. It is an effective and non-destructive sensing method due to its high sensitivity, easy set-up, low background signal, and label-free nature.^95^ ECL does not require any external light sources and there is an alteration of the electrochemical energy into radiative energy by varying the potential at the electrode surface, which shows the promise for the construction of ECL biosensors.^124,125^ These ECL biosensors offers many applications in the field of food, water testing, immunoassay, *etc.*^126^ GQDs were used as sensing probe for the determination of harmful metals or small molecules based on their ECL activities as shown in Table 4.

**Fig. 9:**
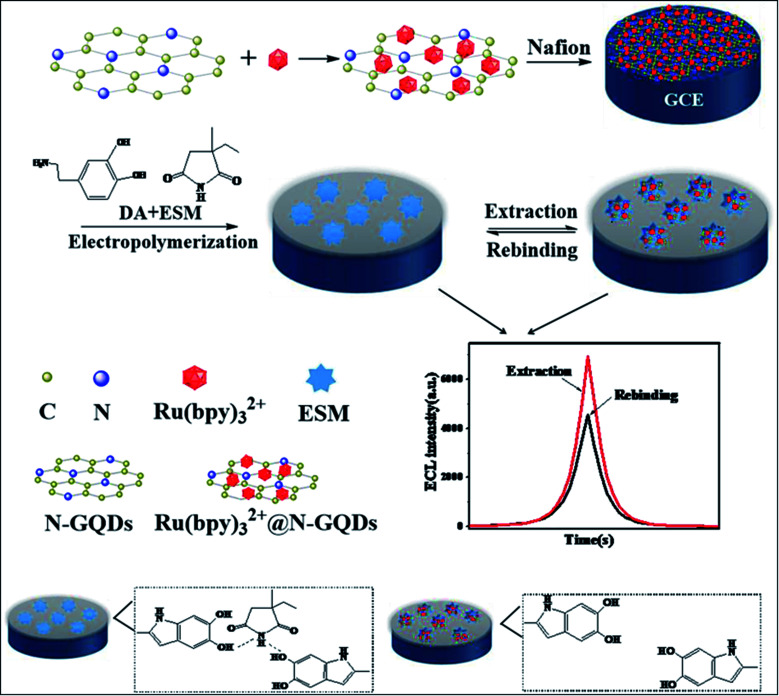
The schematic diagram of MIP ECL sensor for the detection of ethosuximide.^126^ Image is reproduced from ref. 126 with the permission from [Elsevier], copyright [2020].

**Fig. 10:**
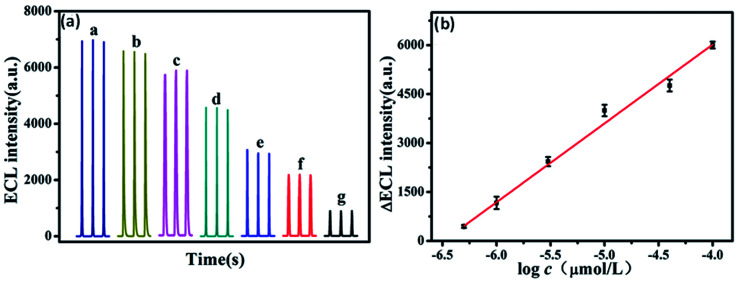
(a) Relative ECL stability curve of MIP ECL sensor with the addition of different concentrations and (b) a calibration graph for the detection of ESM.^126^ Part (a) and (b) are reproduced from ref. 126 with permission from [Elsevier], copyright [2020].

Zhang *et al.*^126^ fabricated Ru(pby)_3_^2+^@N-GQDs [(tris(2,2′-bipyridyl)) ruthenium(ii)@N doped GQDs] by electrostatic interaction between Ru(pby)_3_^2+^ and N-GQDs, which showed excellent and stable ECL properties and acts as a PL sensing probe for the selective determination of ethosuximide (ESM) as shown in Fig. 9. The quantitative relationship among the different concentrations of ESM and variation in the ECL intensity was examined. This is used for reasonable detection in blank plasma and urine samples. Thus, such an efficient and simple MIP on Ru(pby)_3_^2+^@N-GQDs system may be a good model for the fabrication of drug sensors and has a potential application to assay ESM in the biological samples. They found that the ECL intensity was reduced in the presence of different concentrations of ESM and ECL stability curve at each concentration (Fig. 10a). Fig. 10b shows a good linear relationship among Δ*I*_ECL_ (difference of the ECL intensity values in the presence and absence of ESM) and logarithm concentration of ESM with the detection range from 5.00 × 10^−7^ to 1.00 × 10^−4^ mol L^−1^ (Fig. 10b). Peng *et al.*^56^ synthesized water-soluble and uniform-size P, S-GQDs by electrolysis method and demonstrated advancement in ECL properties. The ECL immunosensor was designed for ultra-sensitive detection of okadaic acid (OA) using carboxylated multiwall carbon nanotubes–poly(diallyldimethylammonium) chloride–Au nanocluster (CMCNT-PDDA-AuNCs) composite as a sensing probe to immobilize OA and P, S-GQDs as effective ECL markers. The design of as prepared immunosensor showed high convenience, great sensitivity response, and minor matrix effect. These effective properties of prepared ECL immunosensor used for the OA sensing in mussel samples. Yang *et al.*^127^ fabricated molybdenum disulfide (MoS_2_) GQDs hybrid composite contributing excellent ECL performance. The MoS_2_-GQDs hybrid composite was fabricated as MIP-ECL sensor for the sensitive and selective detection of 2-methyl-4-chlorophenoxyacetic acid. The developed sensor showed great sensitivity, high selectivity, and stability for the detection of pesticides and veterinary drugs in food and environmental safety. Chen *et al.*^128^ synthesized a single luminophor dual-potential ECL system by using N-GQDs as a ratiometric sensor for the efficient detection of Co^2+^ ions. During the cyclic potential scanning, the dissolved oxygen changes to O_2_^−^ and HO_2_^−^ which get involved in the anodic and cathodic producing process of N-GQDs. Co^2+^ ion is used to play the catalytic activity in the intermediate steps of anodic ECL reactions to amplify the anodic intensity and decrease the cathodic ECL intensity due to inhibition and quenching effects on the excited state of the luminophor. The constructed ratiometric dual-potential ECL system shows excellent selectivity against other common metal ions. Utilizing the dual-potential ECL sensor makes the sensing more credible in principle. There have been various sensors developed based on ECL by using GQDs are reported in Table 4.

### 5.3. Electrochemical sensors

GQDs are extensively studied as a kind of electrochemical sensor on account of their exceptional properties such as chemical stability, edge effects, catalytic activity, and good electron mobility.^129^ The electrochemical sensor converts the chemical reactions into electrical signals of the analytes which occur onto the surface of the electrode, thereby providing its surrounding information.^130,131^ The electrochemical approaches are less expensive, highly sensitive, simple operation, and easy to handle, therefore attains extensive applications in the field of medicines, sensing, environmental science/monitoring, and food analysis.^132^ Hence, the electrochemical sensors have gained great interest as compared to current existing sensors in analytical chemistry. The widely used GQDs-based electrochemical sensors are mentioned in Table 4.

**Fig. 11:**
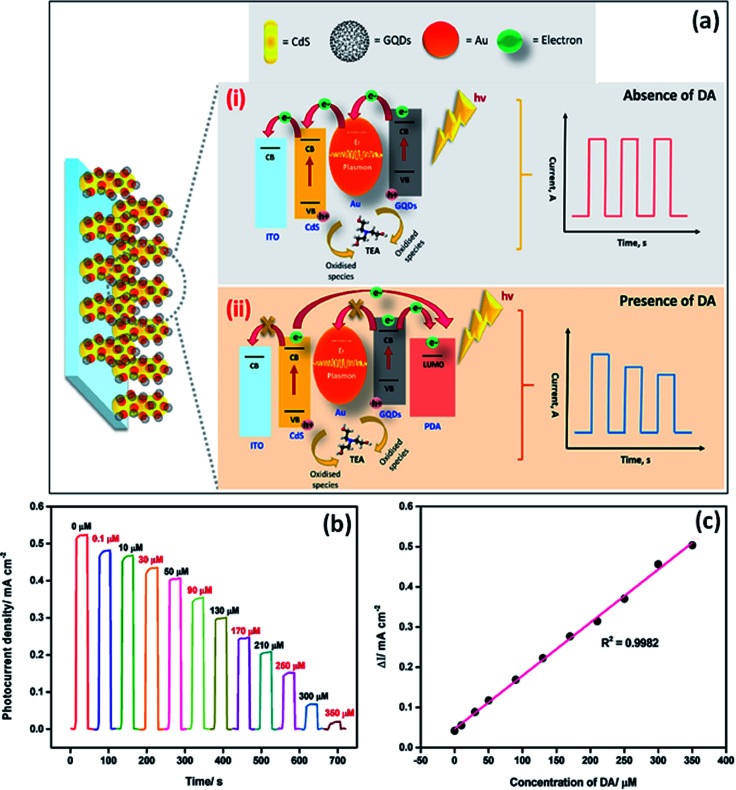
(a) Schematic diagram for charge transfer mechanism of CdS/Au/GQDs composite in (i) absence of dopamine (DA) and (ii) presence of DA, (b) variation in photocurrent response with the addition of different concentration of DA, and (c) calibration plot for the detection of DA by using CdS/Au/GQDs photoelectrode.^133^ Part (a), (b), and (c) are reproduced from ref. 133 with permission from [Elsevier], copyright [2020].

Zhang *et al.*^134^ fabricated MIP/GQDs-Pt nanoparticles (PtNPs)/glassy carbon electrode (GCE) sensing system *via* a simple one-step electro-copolymerization method for highly sensitive detection of trace amount of sulfadimidine (SM_2_) in food. Using the dispersion-corrected (DFT-D) method, a comprehensive computer simulation strategy was used to combine molecular docking and the solvation effect in the screening of bi-functional monomers for the rational design of MIP materials. The sensor is better in terms of effective sensitivity, specificity, stability, ease of operation, and cost. The one-step electro-copolymerization process leads to a robust integration of MIP film with a transducer. Therefore, this method simplifies the preparation of MIP material and lowers the cost. The electro-copolymerization system also offers an important reference for the electro-polymerization reactions and may be extended to supercapacitors and fuel cells. Ibrahim *et al.*^133^ prepared a triple interconnected structure of cadmium sulfide (CdS) modified with Au/GQDs. The introduction of Au and GQDs on the photocatalytic active center of CdS acts as a charge separation mediator and photosensitizer, respectively, which are favorable for charge separation and transportation and PEC conversion. Fig. 11a showed the proposed mechanism of the CdS/Au/GQDs sensor in which CdS/Au/GQDs photoelectrode used for the detection of DA get oxidized to PDA which owns a large number of benzoquinone (BQ) groups (electron-acceptors). The photocurrent responses get reduced with the addition of DA (Fig. 11b). Fig. 11c showed a calibration curve for the sensing of DA with the detection ranging from 0.1 to 350 μM and the value for LOD was 0.0078 μM. Fu *et al.*^135^ synthesized N-GQDs, *via* an electrochemical method for the selective detection of Fe^3+^ ions. Therefore, the distinctive behavior of the heteroatom-doped GQDs provides a novel research platform for real-time applications in environmental safety and control.

## 6. Conclusion and future perspectives

In this review, we have discussed the heteroatom doping (N, P, S, B) of GQDs to advance the chemical, electronic, and optical properties of GQDs. Heteroatom doping can be divided into single heteroatom doping and co-doped heteroatom doping. Both “top-down” and “bottom-up” approaches have been used for developing single heteroatom and co-doped heteroatom GQDs. Recently, the one-step method with a bottom-up approach for the formation of heteroatom doped GQDs has been followed in comparison with the multi-step method which is a long and tedious procedure. It has been reported that the heteroatom doping with larger atomic sizes such as N, S, or smaller atomic sizes such as P, B relative to C exhibited extraordinary properties. The size and surface morphology of different heteroatom doped GQDs were measured by TEM. It was determined that the heteroatom doped GQDs consists of mainly (100) lattice planes that correspond to the graphitic lattice plane. It was found that the heteroatom doped GQDs showed crystalline nature, which was determined by HRTEM, Raman spectroscopy, and XRD pattern. The interlayer spacing changed with heteroatom doping as compared to the interlayer spacing of graphite. The heteroatom doped GQDs have gained improvement in their intrinsic properties like chemical activity, electronic structure, band gap, and PL quantum yield. Heteroatom doping tune the energy band gap of GQDs which modifies their absorption wavelength and PL emission wavelength. The electronegativity difference of heteroatoms as compared to carbon atoms play a great role in tuning its electronic structure. Heteroatom doping of GQDs improves their optical, ECL, and electrochemical properties that result in the development of smart sensing probes for the sensing of different metal ions, small organic molecules, macromolecules (like DNA, RNA, proteins), insecticides, and pesticides.

Nowadays, heteroatom doping of GQDs has developed as an appropriate approach for improving the properties of GQDs. But heteroatom doped GQDs still have some limitations such as they are mostly blue or green emissive under UV light irradiation, decrease in the PL intensity when emission peak is shifted towards higher wavelength and purification of GQDs. Also, the synthesis of uniform-sized GQDs is still a challenge. Mostly the reported methods give short scale-production of heteroatom doped GQDs but there is a need to produce them on a large scale. Further, the synthesis of heteroatom doped GQDs by simple methods and efficient purification techniques with different sizes is in great demand at the current time. The PL mechanism of heteroatom doped GQDs is still in debate. So, the theoretical calculations can provide an approach to understand the exact PL mechanism. Heteroatom doped GQDs have been used as various types of sensors such as PL, ECL, and electrochemical sensors based on different mechanisms. These sensors showed many advantages over the traditional sensors. But some of the sensors based on heteroatom GQDs showed drawbacks, for example, long fabrication process, pH and temperature dependent detection, long incubation time, complex process, *etc.* The removal of these drawbacks has become a future challenge. By eliminating these obstacles, the heteroatom doped GQDs become more prominent in the various fields.

## Author contributions

Neeraj Sohal: formatted the original draft, surveyed the related literature, analyzed the reported data, and co-wrote the paper. Banibrata Maity: supervised, set the format of the paper, and co-wrote the paper. Soumen Basu: supervised, formatted the original draft, and co-wrote the paper.

## Conflicts of interest

The authors don't have any conflict of interest in the publication of the manuscript.

## Supplementary Material
